# Efficient n‐Doping of Organic Semiconductors via a Broadly Applicable Nucleophilic‐Attack Mechanism

**DOI:** 10.1002/advs.202520487

**Published:** 2025-11-06

**Authors:** Huan Wei, Tong Wu, Chuanding Dong, Chen Chen, Zhenqi Gong, Jiangnan Xia, Chengyuan Peng, Jiaqi Ding, Yu Zhang, Wenpei Shi, Stefan Schumacher, Xue Zhang, Yugang Bai, Lang Jiang, Lei Liao, Thuc‐Quyen Nguyen, Yuanyuan Hu

**Affiliations:** ^1^ Changsha Semiconductor Technology and Application Innovation Research Institute College of Semiconductors (College of Integrated Circuits) Hunan University Changsha 410082 China; ^2^ International Science and Technology Innovation Cooperation Base for Advanced Display Technologies of Hunan Province School of Physics and Electronics Hunan University Changsha 410082 China; ^3^ Engineering Research Center for Nanomaterials Henan University Kaifeng 475004 China; ^4^ State Key Laboratory of Chemo/Biosensing and Chemometrics College of Chemistry and Chemical Engineering Hunan University Changsha 410082 China; ^5^ Department of Physics and Center for Optoelectronics and Photonics Paderborn (CeOPP) Paderborn University Warburger Strasse 100 33098 Paderborn Germany; ^6^ Science and Technology on Advanced Ceramic Fibers and Composites Laboratory College of Aerospace Science and Engineering National University of Defense Technology Changsha 410000 China; ^7^ Spin‐X Institute School of Microelectronics South China University of Technology Guangzhou 511442 China; ^8^ College of Chemical Engineering Hebei University of Technology Tianjin 300401 China; ^9^ Center for Polymers and Organic Solids Department of Chemistry and Biochemistry University of California at Santa Barbara Santa Barbara CA 93106 USA

**Keywords:** conductivity, doping efficiency, doping mechanism, nucleophilic‐attack, organic semiconductors

## Abstract

The development of efficient and broadly applicable n‐doping strategies for organic semiconductors (OSCs) is crucial for advancing the performance of various organic electronic devices. Here, a novel nucleophilic‐attack n‐doping mechanism is unveiled that achieves exceptionally high conductivity in doped OSC films and demonstrates broad applicability across OSCs. The remarkable efficacy of n‐Butyl lithium (n‐BuLi) is highlighted in n‐doping C_60_ and PC_61_BM, achieving a conductivity of 1.27 S cm^−1^ and 2.57 S cm^−1^, respectively, which are among the highest reported values for these materials. The investigation reveals that the n‐BuLi anion interacts with electron‐deficient units in OSCs, generating a carbanion that facilitates efficient electron transfer for n‐doping. This mechanism is further validated across diverse fullerenes, polymeric, and small molecule OSCs, and is extendable to other high‐performance dopants such as tert‐Butyllithium (tert‐BuLi) and sodium ethoxide (NaOEt). Device studies show that n‐BuLi‐doped C_60_ enables substantially improved diode rectification, attributed to greater junction built‐in potential. These findings establish a unified chemical‐bonding‐based n‐doping paradigm, complementing existing electrophilic‐attack p‐doping concepts, and pave the way for achieving efficient doping of OSCs for advanced organic electronic applications.

## Graphical abstract

A novel n‐doping mechanism for organic semiconductors (OSCs) is revealed, based on a nucleophilic‐attack reaction. Using n‐Butyl lithium (n‐BuLi) as a model dopant, this strategy achieves exceptionally high conductivity in C_60_ and PC_61_BM. The mechanism is broadly applicable to various OSCs and other nucleophilic dopants, establishing a new chemical‐bonding paradigm for efficient n‐doping OSCs.

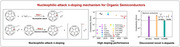

## 1 Introduction

Doping is a crucial technique for a wide range of organic semiconductor (OSC) device applications, ranging from organic light‐emitting diodes (OLEDs),^[^ ^1^, ^2^, ^3^ ^]^ organic photovoltaics (OPVs),^[^ ^4^, ^5^, ^6^ ^]^ and organic thermoelectrics (OTEs)^[^ ^7^, ^8^, ^9^ ^]^ to organic field‐effect transistors (OFETs).^[^ ^10^, ^11^, ^12^ ^]^ While significant progress has been made in p‐doping methodologies,^[^ ^13^, ^14^, ^15^, ^16^, ^17^, ^18^ ^]^ efficient and broadly applicable n‐doping of OSCs remains a persistent challenge, hindering the advancement of next‐generation organic electronic technologies.

The most established n‐doping approaches rely on strong reducing agents, including organic and organometallic dimers, which operate via straightforward one‐electron transfer (ET) mechanisms.^[^ ^19^, ^20^, ^21^ ^]^ These dopants are designed to transfer electrons to the lowest unoccupied molecular orbital (LUMO) of the OSC, creating radical anions that enhance conductivity. However, as highlighted in recent reviews, this approach faces significant limitations.^[^ ^22^ ^]^ The elevated highest occupied molecular orbital (HOMO) levels required for efficient electron transfer render the synthesis of stable reducing dopants challenging.^[^ ^23^, ^24^ ^]^ Furthermore, the straightforward ET mechanism is inherently limited in scope, as it requires a precise alignment between the HOMO of the dopant and the LUMO of the OSC, restricting its applicability to a narrow range of OSCs.

Alternative n‐doping methods, including hydride transfer with dopants such as 4‐(1,3‐dimethyl‐2,3‐dihydro‐1H‐benzimidazole‐2‐yl)phenyl)dimethylamine (N‐DMBI), have shown promise in overcoming some of the limitations of straightforward ET doping. However, these dopants often suffer from solubility issues, phase separation when mixed with OSCs, and limited doping efficiency, typically below 10%, which limit their broader application.^[^ ^25^, ^26^, ^27^ ^]^ Additionally, organic superbases like 1‐*tert*‐Butyl‐2,2,4,4,4‐pentakis(dimethylamino)‐2λ^5^,4λ^5^‐catenadi(phosphazene) (P2‐t‐Bu) and 1‐*tert*‐Butyl‐4,4,4‐tris(dimethylamino)‐2,2‐bis[tris(dimethylamino)‐phosphoranylidenamino]‐2λ^5^,4λ^5^‐catenadi(phosphazene) (P4‐t‐Bu) have been explored as n‐dopants, which are suggested to operate via a deprotonation‐induced n‐doping mechanism.^[^ ^22^, ^28^, ^29^ ^]^ While these approaches expand the range of dopable OSCs, they often require specific structural features in the OSC, such as acidic protons, limiting their universality. Recent studies have suggested complex doping mechanisms—those involving multistep reactions or intermediates—may overcome some constraints of direct ET.^[^ ^22^ ^]^ Despite these advancements, the field still lacks a broadly applicable and efficient n‐doping mechanism that can address the limitations of existing approaches.

In this context, we report here the discovery of a new n‐doping pathway based on nucleophilic‐attack chemistry, using n‐butyllithium (n‐BuLi) as a model dopant. While organolithium reagents like n‐BuLi have been used as powerful lithiation agents for structural phase engineering in inorganic 2D materials,^[^ ^30^ ^]^ their role and underlying mechanism as molecular n‐dopants for OSCs through covalent chemical reactions have, to our knowledge, remained unexplored. We demonstrate that n‐BuLi can efficiently n‐dope C_60_, achieving a conductivity of 1.27 S cm^−1^, among the highest ever for n‐doped C_60_. Mechanistic studies reveal that n‐BuLi covalently bonds to electron‐deficient units in OSCs, generating carbanion intermediates that drive highly efficient and stable n‐doping. Remarkably, this mechanism is broadly applicable across different OSC systems and can guide the identification of other high‐performance n‐dopants, such as tert‐Butyllithium (tert‐BuLi) and sodium ethoxide (NaOEt). Our work establishes a unified chemical‐bonding doping paradigm, alongside previous electrophilic‐attack p‐doping concepts, that paves the way for rational design and development of high‐performance n‐doped OSCs.

## 2 Results and Discussion

### 2.1 Doping Effect of n‐BuLi in C_60_

**Figure 1:**
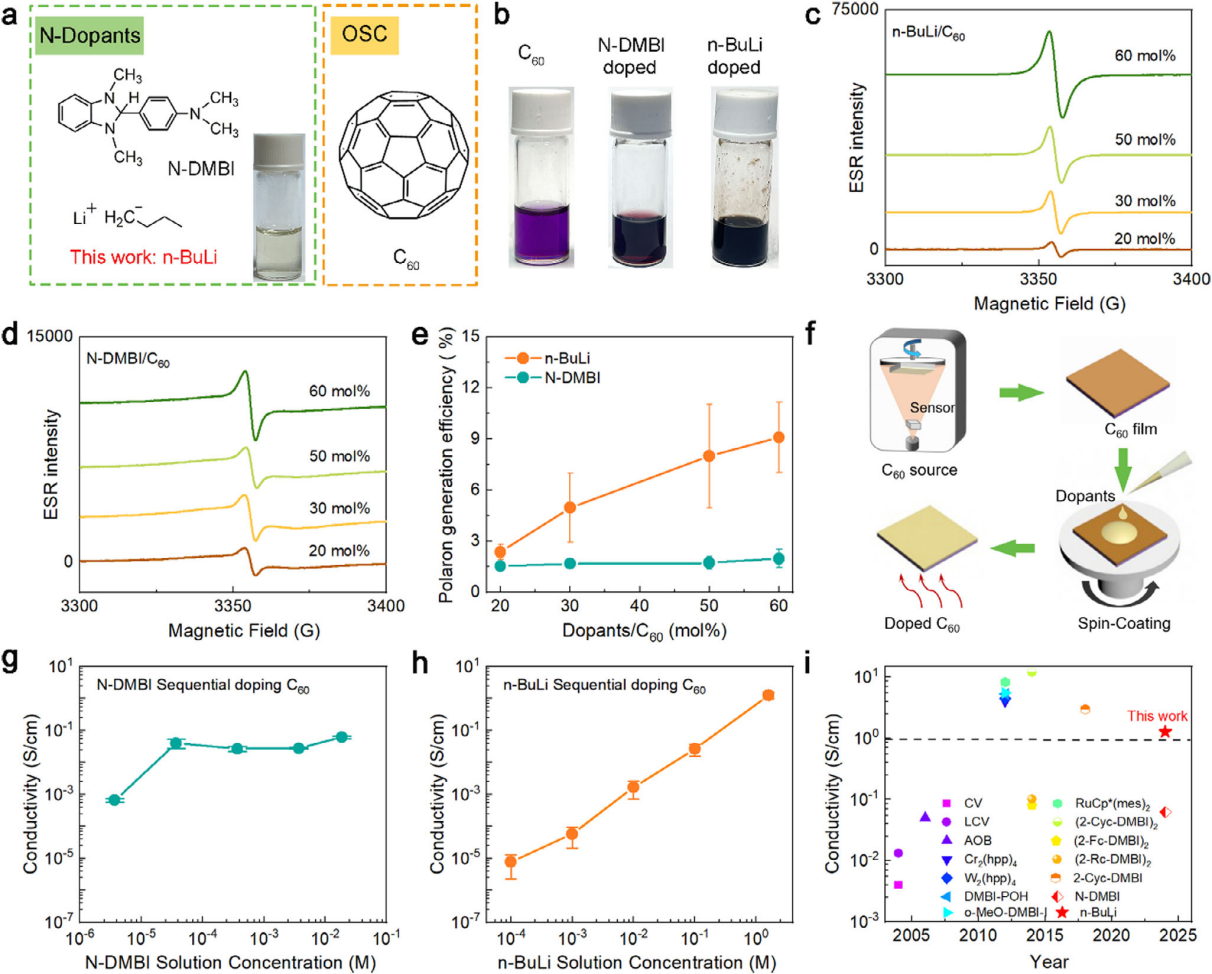
Investigations of the n‐doping effect of n‐BuLi. Molecule structures of a) dopants n‐BuLi and N‐DMBI, and n‐type OSC C_60_. b) Color change of undoped, N‐DMBI‐ and n‐BuLi‐doped C_60_ solution. The ESR spectroscopy of c) n‐BuLi‐ and d) N‐DMBI‐doped C_60_ films as a function of doping concentration measured at room temperature. e) The polaron generation efficiency of n‐BuLi and N‐DMBI as a function of doping concentration. The polaron generation efficiency values are the mean of two independent replicate measurements, and the errors are the corresponding standard deviation. f) Schematic diagrams for the sequential doping method. The conductivity of C_60_ doped by g) N‐DMBI and h) n‐BuLi using the sequential doping method. The conductivity values are the mean of three samples, and the errors are the corresponding standard deviation. i) The comparison of the conductivity values of doped C_60_ with previously reported values.

The chemical structure of the dopants n‐BuLi and N‐DMBI are depicted in **Figure** **1**a. n‐BuLi consists of lithium cations and n‐butyl anions and is a liquid at room temperature, displaying good solubility in common organic solvents such as chlorobenzene (CB), hexane, acetonitrile (ACN), and toluene (TL) (Figure S1, Supporting Information). While recognizing that n‐BuLi is a highly reactive compound requiring inert‐atmosphere handling, its potent and unambiguous nucleophilicity makes it an ideal candidate for investigating the new doping mechanism. We selected C_60_ as a model host semiconductor to evaluate the doping effect of n‐BuLi. Although C_60_ generally exhibits limited solubility in organic solvents, it can be dissolved in CB with a solubility below 7 mg mL^−1^.^[^ ^31^ ^]^ As illustrated in Figure 1b, the initial C_60_ solution appears light purple. Upon addition of n‐BuLi, the solution color transitions from purple to dark, similar to that of the mixture N‐DMBI with the C_60_ solution, indicating the occurrence of doping.

Subsequently, electron spin resonance (ESR) measurements were conducted on both the pristine C_60_ and n‐BuLi:C_60_ solids. The n‐BuLi: C_60_ samples display a pronounced, concentration‐dependent paramagnetic ESR signal, which is absent in pristine C_60_, indicating efficient polaron formation and successful doping (see Figure 1c; Figure S2a, Supporting Information). Using N‐DMBI as a reference dopant, we found that n‐BuLi produces a notably stronger ESR signal at equivalent doping concentrations (see Figure 1d), underscoring its high doping efficiency. The number of polarons and the polaron generation efficiency (η_i_)—defined as the ratio of polarons to dopant molecules—were quantified from ESR data (Figure S2b, Supporting Information). As illustrated in Figure 1e, η_i_ for n‐BuLi increases with doping concentration, reaching ≈8% at 60 mol%, which, while modest, is still 2–5 times greater than that achieved with N‐DMBI.

We next examined the effect of doping on the electrical conductivity of C_60_ films. Due to the limited solubility of C_60_, which complicates the formation of uniform films via spin coating, we employed a sequential doping method. This involved thermally evaporating C_60_ films followed by spin‐coating the dopant solution on top, as illustrated in Figure 1f (see more details in the Figure S3, Supporting Information). The doping levels were adjustable by controlling the concentration of the dopant solution. Figure 1g,h show that n‐BuLi‐doped films reach a maximum conductivity of 1.27 S cm^−1^, more than twenty times higher than films doped with N‐DMBI (< 0.1 S cm^−1^) (see more details in Figures S4 and S5, Supporting information). Notably, n‐BuLi achieves a conductivity of 1.27 S cm^−1^, among the highest ever for n‐doped C_60_, as indicated in Figure 1i (see Table S1, Supporting Information).

### 2.2 Revealing the Nucleophilic‐Attack Doping Mechanism

Having established the effective n‐doping of C_60_ by n‐BuLi, we sought to elucidate the underlying mechanism. n‐BuLi, widely utilized in organic synthesis, is known for its strong base and nucleophilic properties.^[^ ^32^ ^]^ Typically, n‐BuLi can engage in two types of reactions with other substances: nucleophilic‐attack reactions and deprotonation reactions.^[^ ^33^, ^34^, ^35^, ^36^, ^37^ ^]^ As C_60_ lacks hydrogen, the possibility of a deprotonation reaction is excluded. Thus, we hypothesized the involvement of a nucleophilic‐attack mechanism, in which the nucleophilic carbon of n‐BuLi attacks a carbon atom on the fullerene cage, leading to the formation of a butylated fullerene derivative.

**Figure 2:**
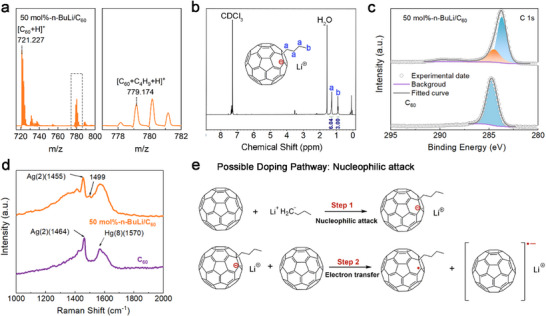
Evidence and schematic illustration for the nucleophilic attack n‐doping mechanism. a) MALDI‐TOF MS spectrum of the sample containing 50 mol% n‐BuLi‐doped C_60_. b) ^1^H NMR characterization of n‐BuLi‐doped C_60_ in CDCl_3_. c) The XPS spectra of C 1s in C_60_ and 50 mol% n‐BuLi‐doped C_60_ films. d) The Raman spectrum of C_60_ (purple line) and 50 mol% n‐BuLi‐doped C_60_ (orange line) films. e) Proposed n‐doping mechanism of n‐BuLi and C_60_.

To verify this hypothesis, matrix‐assisted laser desorption/ionization time‐of‐flight mass spectroscopy (MALDI‐TOF MS) was used to identify the products. This technique enables sensitive detection of analytes in a sample and provides an accurate measurement of the mass‐to‐charge ratio (m/z), from which the chemical composition of each analyte is determined. As shown in **Figures** **2**a and S6 (Supporting Information), distinct peaks including C_60_, [C_60_+H]^+^ at m/z 721.227, and a with [C_60_+C_4_H_9_+H]^+^ peak at m/z 779.174, indicates the chemical attachment of the n‐BuLi anion (C_4_H_9_^−^) to C_60_, confirming nucleophilic attack. Proton nuclear magnetic resonance (^1^H NMR) as further used to verify the butyl attachment. The product, washed to remove excess dopant and dissolved in CDCl_3_, indicated the presence of the butyl group by showing peaks ≈1 ppm, and the integration values of the peaks were also consistent with the addition of butyl groups (see Figure 2b; Figure S7, Supporting Information).

Further evidence comes from X‐ray photoemission spectroscopy (XPS). In the pristine C_60_ sample, the C 1s peak is distinct and appears at a binding energy of 284.5 eV (Figure 2c), characteristic of sp^2^‐hybridized carbon in a C═C environment.^[^ ^38^, ^39^ ^]^ The C 1s spectrum of the 50 mol% n‐BuLi‐doped C_60_ film reveals a new peak at 285.5 eV, attributed to the introduction of sp^3^‐hybridized carbon atoms (C─C single bonds) due to the addition of the n‐Butyl group (see more results in Figure S8, Supporting Information). Raman spectroscopy of the films (Figure 2d) shows the Ag(2) mode of C_60_ shifts from 1464 to 1455 cm^−1^ and a new peak at 1499 cm^−1^ appears, assigned to C─H bending, both confirming butyl group incorporation onto C_60_.^[^ ^40^ ^]^

Putting these data together, we propose the following mechanism: n‐BuLi attacks the electron‐deficient carbons of C_60_, forming butylated fullerene derivatives and generating a fulleride anion. This anion has a high electron density and readily transfers an electron to a neutral C_60_ molecule, producing the radical anion (C_60_^−•^) (Figure 2e).^[^ ^22^ ^]^ This C_60_ radical anion is stabilized by the delocalization of the extra electron across the entire fullerene cage, making it relatively stable compared to other radical species. Notably, this nucleophilic‐attack n‐doping pathway echoes our previously reported p‐doping mechanism based on electrophilic‐attack (with Ph_3_C⁺),^[^ ^41^ ^]^ complementing the mechanistic understanding of chemical bonding‐based OSC doping.

### 2.3 General Applicability of Nucleophilic‐Attack Doping to Other OSCs

**Figure 3:**
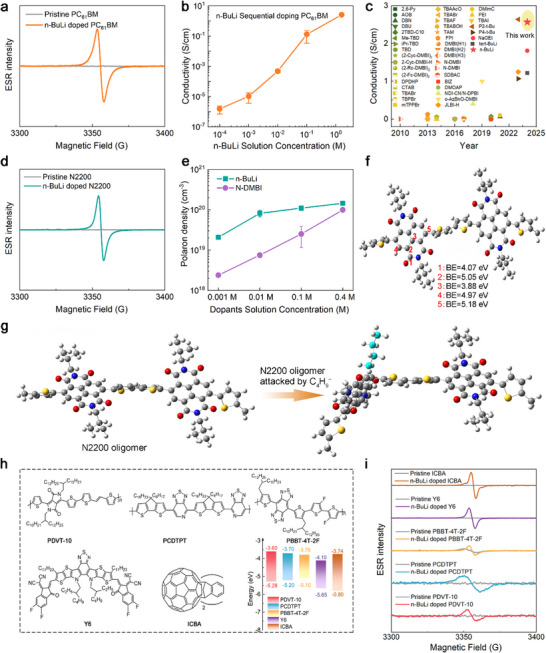
Characterizations on the capability of n‐doping different OSCs by n‐BuLi. The ESR spectra of n‐BuLi‐doped a) PC_61_BM and d) N2200 solutions. b) The conductivity of PC_61_BM doped by n‐BuLi using the sequential doping method. The conductivity values are the mean of three samples, and the errors are the corresponding standard deviation. c) The conductivity values of doped PC_61_BM reported in the literature. e) The polaron density of n‐BuLi‐ and N‐DMBI‐doped N2200 films. The polaron density values are the mean of two independent replicate measurements, and the errors are the corresponding standard deviation. f) The theoretical calculations of nucleophilic‐attack reaction between the n‐BuLi anion and the NDI unit. g) The changes in the polymer backbone after nucleophilic‐attack reaction of n‐BuLi anion and NDI unit. h) The molecule structures and energy levels of PDVT‐10, PCDTPT, PBBT‐4T‐2F, Y6, and ICBA. i) The ESR spectroscopy of pristine and n‐BuLi‐doped OSCs.

Building on these insights, we investigated whether the nucleophilic‐attack mechanism extends to other OSCs featuring acceptor units. First, in the n‐BuLi: PC_61_BM system, we observed a pronounced color change in solution (from dark red to even darker, Figure S10, Supporting Information), as well as distinct ESR signals (**Figure** **3**a; Figure S11, Supporting Information), indicating successful doping. MALDI‐TOF MS further established the formation of [PC_61_BM+C_4_H_9_]^+^, confirming nucleophilic attack (Figure S12, Supporting Information). However, it is important to note that n‐BuLi is also a strong superbase, which may lead to deprotonation reactions in PC_61_BM. Such deprotonation can also induce n‐doping of OSCs, as we have reported previously.^[^ ^28^ ^]^ While we have confirmed the occurrence of nucleophilic‐attack reactions, we cannot exclude the possibility of deprotonation. Thus, it is plausible that two doping pathways coexist in the n‐BuLi: PC_61_BM system.

The conductivity of n‐BuLi‐doped PC_61_BM films, prepared using a sequential doping method, increases sharply with n‐BuLi doping, peaking at (2.57 ± 0.06) S cm^−1^ at 1.6 m n‐BuLi (Figure 3b; Figures S13 and S14, Supporting Information). Figure 3c summarizes the conductivity values for PC_61_BM doped with various dopants, with n‐BuLi achieving among the highest values currently reported for n‐doped PC_61_BM (refer to Table S2, Supporting Information), confirming its high‐performance as an n‐dopant.

We further employed n‐BuLi to dope N2200, a polymer semiconductor based on naphthalene diamide (NDI) acceptor units that has been extensively studied as a leading n‐type polymer semiconductor.^[^ ^42^, ^43^, ^44^ ^]^ ESR measurements of n‐BuLi‐doped N2200 solutions revealed pronounced paramagnetic signals indicative of polaron generation (Figure 3d), confirming the doping effect. Notably, the polaron density in n‐BuLi‐doped N2200 films was found to be significantly higher than that in N‐DMBI‐doped films at comparable doping ratios (Figure 3e; see more information in Figure S15, Supporting Information), suggesting the remarkable doping efficiency of n‐BuLi. Nevertheless, the conductivity of doped N2200 was observed to be lower than that achieved with N‐DMBI (see more information in Figure S16, Supporting Information). Theoretical calculations suggest that the nucleophilic‐attack reaction between the n‐BuLi anion and the carbon atom at position 5 in the NDI unit is energetically favorable (see Figure 3f), with a binding energy (BE) of −5.18 eV. This reaction, however, induces significant bending of the polymer backbone (see Figure 3g), which likely deteriorates charge transport efficiency and limits conductivity enhancement. These findings not only confirm the efficient doping induced by nucleophilic‐attack reaction, but also highlight the interplay between dopant bonding and polymer backbone configuration, providing insights for the future design and optimization of nucleophilic n‐dopants.

To test the chemical generality of the nucleophilic‐attack mechanism, we extended our study to a diverse range of OSCs containing different electron‐acceptor units. These included the D‐A copolymers PDVT‐10 (DPP acceptor),^[^ ^45^ ^]^ PCDTPT (PT acceptor),^[^ ^46^ ^]^ and PBBT‐4T‐2F (BBT acceptor),^[^ ^12^, ^47^, ^48^ ^]^ as well as the renowned non‐fullerene acceptor Y6^[^ ^49^, ^50^ ^]^ and the fullerene derivative ICBA.^[^ ^51^ ^]^ Despite the significant variation in the chemical structures and energy levels of these materials (Figure 3h), mixing each with n‐BuLi consistently produced distinct solution color changes and strong ESR polaron signals (Figure 3i; Figure S17, Supporting Information). This result confirms that the nucleophilic‐attack reaction is not limited to a specific OSC but is a chemically versatile mechanism applicable to a wide array of materials featuring electron‐deficient moieties. It is important to note, however, that polaron generation is only the first step in the doping process. As demonstrated by the N2200 case, achieving high conductivity also requires that the subsequent charge transport is not impeded by factors such as dopant‐induced backbone configuration or morphological changes. Therefore, while this study establishes the broad chemical applicability of nucleophilic‐attack doping, further investigation would be required to optimize the final electronic performance for each specific OSC.

### 2.4 Exploration of Other Nucleophilic‐Attack n‐Dopants

**Figure 4:**
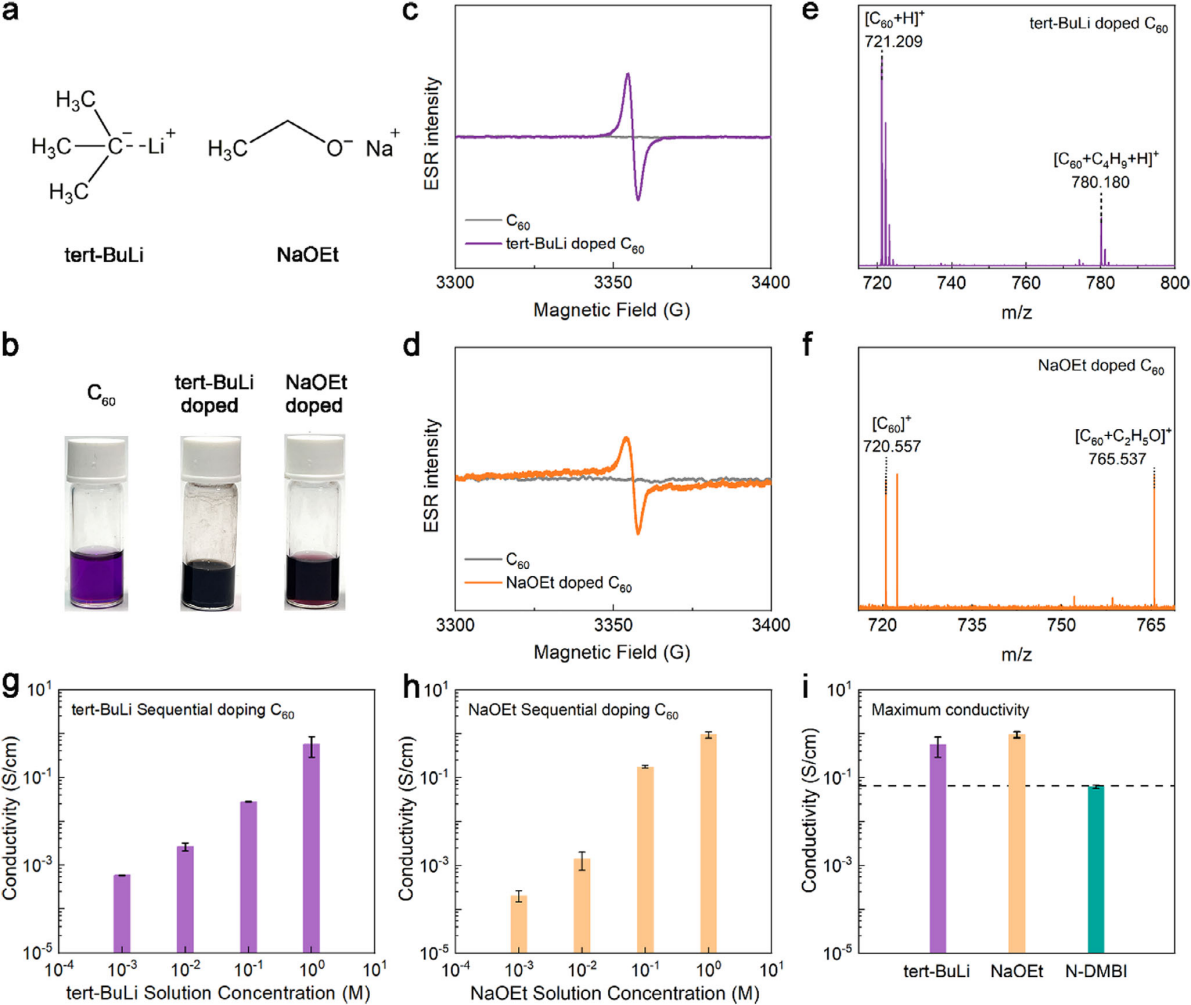
Characterization of novel n‐dopants identified through the nucleophilic‐attack n‐doping mechanism. a) Molecule structures of dopants tert‐BuLi and NaOEt. b) Color change of undoped, tert‐BuLi‐ and NaOEt‐doped C_60_ solution. The ESR spectroscopy of c) tert‐BuLi‐ and d) NaOEt‐doped C_60_. MALDI‐TOF MS spectrum analysis of e) tert‐BuLi‐ and f) NaOEt‐doped C_60_. Electrical conductivity of C_60_ doped by g) tert‐BuLi‐ and (h) NaOEt as a function of doping concentration. i) Comparison of the maximum conductivity values of tert‐BuLi‐, NaOEt‐, and N‐DMBI‐doped C_60_. The conductivity values are the mean of three samples, and the errors are the corresponding standard deviation.

Importantly, our mechanistic insights guided the search for additional high‐performance n‐dopants. tert‐BuLi, structurally similar to n‐BuLi, was shown to efficiently dope C_60_ as well (**Figure** **4**a; Figure S18, Supporting Information). When tert‐BuLi is mixed with C_60_, the solution changes from purple to dark, as shown in Figure 4b. ESR measurements reveal a strong paramagnetic signal (Figure 4c), indicating successful doping of C_60_ by tert‐BuLi. MALDI‐TOF MS identifies the [C_60_+C_4_H_9_+H]^+^ product at m/z peak 780.180 (Figure 4e), verifying the nucleophilic‐attack mechanism.

We also identified NaOEt, a classic nucleophile, as an effective n‐dopant. Notably, unlike n‐BuLi or tert‐BuLi, which are nucleophiles and also superbases, NaOEt has significantly weaker basicity. Unlike n‐BuLi and tert‐BuLi, NaOEt is a much weaker base and acts more selectively as a nucleophile. When mixed with C_60_, a color change and distinct ESR signal are observed (Figure 4b,d). MALDI‐TOF MS confirms the formation of [C_60_+C_2_H_5_O]^+^ at m/z 765.537, which indicate the chemical attachment of the NaOEt (C_2_H_5_O^−^) anions to C_60_, again confirming nucleophilic‐attack doping (Figure 4f).

The conductivity of C_60_ films doped with tert‐BuLi and NaOEt reached maxima of (0.57 ± 0.28) S cm^−1^ (1.3 m tert‐BuLi) and (0.96 ± 0.15) S cm^−1^ (1.0 m NaOEt), as shown in Figure 4g,h, respectively (see more results in Figures S19 and S20, Supporting Information). Both values are substantially higher than with conventional n‐dopants such as N‐DMBI (Figure 4i). These dopants are also highly effective for PC_61_BM, raising its conductivity from ≈10^−7^ S cm^−1^ in the pristine films to (1.22 ± 0.62) S cm^−1^ for tert‐BuLi and (1.81 ± 1.39) S cm^−1^ for NaOEt (Figures S21–S24, Supporting Information).

Interestingly, for both C_60_ and PC_61_BM, the observed trend in conductivity follows the same order: n‐BuLi results in the highest conductivity, tert‐BuLi yields the lowest conductivity, and NaOEt falls in between. These differences can be attributed to the interplay between the structural and electronic properties of the dopants. The linear alkyl chain of n‐BuLi minimizes steric hindrance, facilitating efficient nucleophilic attack and uniform doping. In contrast, the bulky tert‐butyl group in tert‐BuLi introduces significant steric hindrance, reducing reaction efficiency and dopant diffusion. NaOEt, with its smaller ethoxide ion, achieves intermediate conductivity by balancing steric hindrance and nucleophilic efficiency. These results underscore the critical importance of dopant molecular design in optimizing the doping process. Reduced steric hindrance and enhanced nucleophilic‐attack efficiency of the dopants are key factors for achieving high conductivity in n‐doped OSCs.

### 2.5 n‐BuLi‐doped C_60_ Films for High‐Rectification Ratio Diode Devices

**Figure 5:**
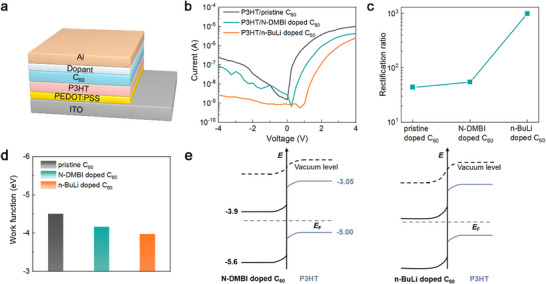
Characterization of organic diodes based on P3HT/doped C_60_. a) Schematic device structure of the P3HT/doped C_60_ diode. b) Current versus voltage (*I*‐*V*) curves of the organic diode with different n‐doped C_60_. c) The rectification ratio calculated from the *I*‐*V* curves of (b). d) The work function of pristine C_60_ and C_60_ films doped with different dopants. e) The schematic diagram shows the band bending at the P3HT/C_60_ interface with different dopants.

To demonstrate the application potential, we constructed organic diodes using PN heterojunctions of p‐type P3HT and n‐type doped C_60_ films (**Figure** **5**a). The doping concentration was optimized for each dopant to maximize conductivity. The current–voltage (*I*–*V*) characteristics (Figure 5b) show that n‐BuLi‐doped C_60_ devices exhibit pronounced rectification, much stronger than devices based on either pristine C_60_ or N‐DMBI‐doped C_60_. The rectification ratio is defined as the absolute value of the current ratio at +4 V and −4 V. As shown in Figure 5c, the n‐BuLi‐doped device exhibits a rectification ratio of ≈10^3^, more than one order of magnitude higher than for N‐DMBI‐based devices.

The higher rectification ratio is attributed to the larger built‐in potential across the PN junction formed with n‐BuLi doped C_60_. Analysis via Kelvin probe shows the work function of C_60_ drops from −4.50 eV (pristine) to −4.17 eV (N‐DMBI‐doped) and further to −3.98 eV (n‐BuLi‐doped), reflecting an upward shift of the Fermi level (*E*_F_) and increased carrier density (Figure 5d; Figure S25, Supporting Information).^[^ ^52^ ^]^ Schematic diagram of the band bending at the P3HT/doped C_60_ interface is illustrated in Figure 5e, which shows greater band bending at the interface for the more strongly doped C_60_, confirming enhanced junction built‐in potential and device performance.^[^ ^53^, ^54^ ^]^

### 2.6 Revisiting Doping Mechanisms: From Redox to Chemical‐bonding Approaches

A fundamental goal in the field is to develop advanced doping strategies. While redox‐based n‐doping has been well established, it suffers from reliance on energy‐level alignment and often low efficiency unless additional measures like ion‐exchange^[^ ^14^, ^55^, ^56^, ^57^ ^]^ or catalysts^[^ ^58^, ^59^ ^]^ are employed. In view of these challenges, exploring and revealing novel doping mechanisms is crucial for breakthroughs in the field.

**Figure 6:**
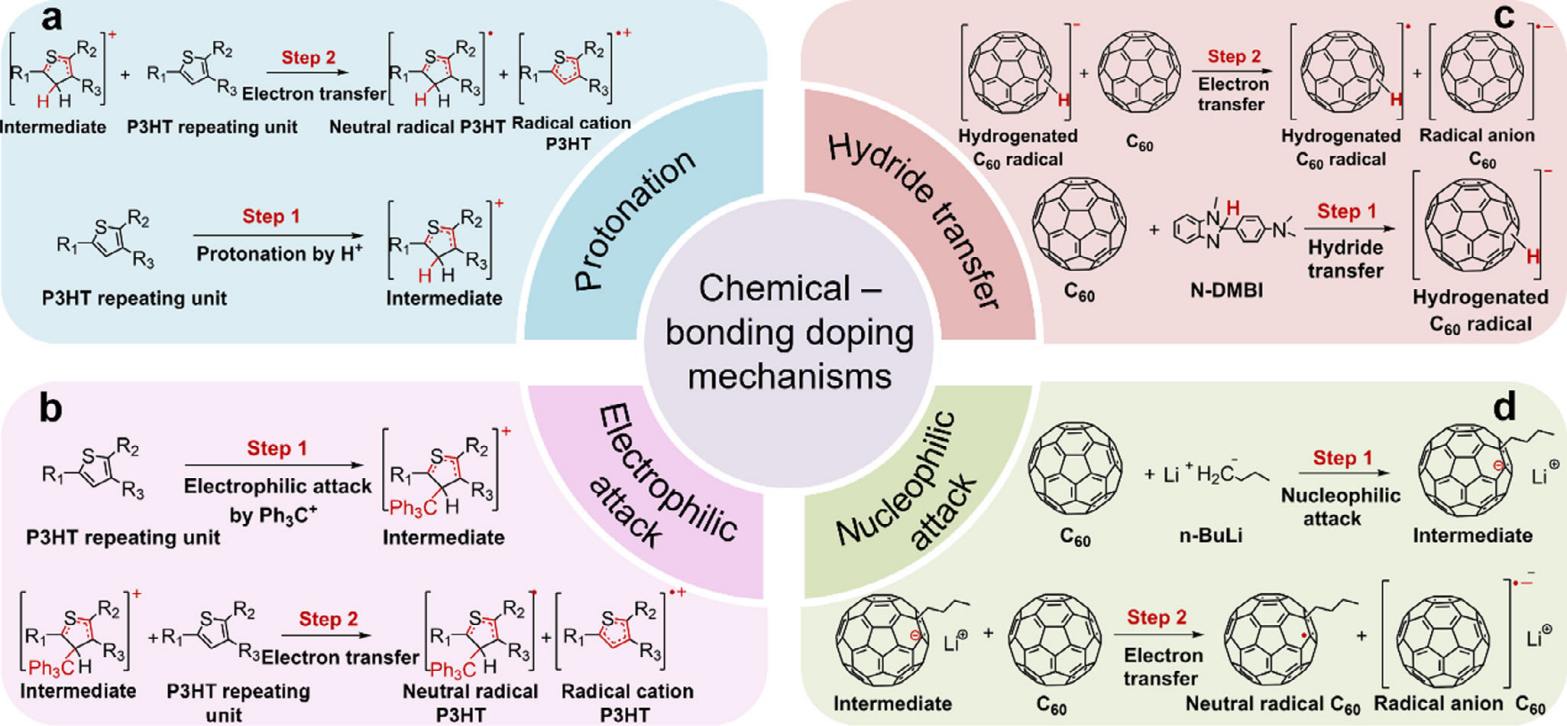
Summary of chemical‐bonding based doping mechanisms. a) Protonation and b) electrophilic‐attack doping mechanism for p‐doping. c) Hydride transfer and d) nucleophilic‐attack doping mechanism for n‐doping.

As depicted in **Figure** **6**a, the protonation doping mechanism, which involves the protonation of OSCs with acidic dopants, resulting in p‐doping, has been identified.^[^ ^17^ ^]^ Complementing this, the hydride transfer doping mechanism, based on the attachment of a hydride ion to the conjugated backbone of OSCs, was proposed and verified (Figure 6c).^[^ ^22^, ^60^, ^61^, ^62^ ^]^ In summary, the protonation p‐doping mechanism involves reaction with H^+^ (proton) while the hydride transfer doping mechanism involves reaction with H^−^ (hydride ion).

Recently, we revealed a p‐doping mechanism based on the electrophilic‐attack reaction between dopants and OSCs (Figure 6b).^[^ ^41^, ^63^ ^]^ A typical example is the use of the trityl cation, a strong electrophile that forms chemical bonds with electron‐rich units in OSCs, such as thiophenes and benzenes. This mechanism provides an efficient and broadly applicable p‐doping strategy. Notably, protonation reaction can be viewed as a specific case of electrophilic‐attack, where the electrophile is H^+^.

The current work establishes a mechanistically analogous n‐doping pathway based on nucleophilic‐attack: nucleophiles reacting covalently with OSCs to enable efficient and chemically general n‐doping (Figure 6d). Conceptually, hydride‐transfer is a subset of nucleophilic‐attack, since H^−^ is a nucleophile. Therefore, our findings complete the landscape of chemical bonding‐based doping mechanisms for OSCs, which contrast with non‐bonding redox reactions and offer general routes for finely‐tuned doping and improved device performance.

## 3 Conclusion

In conclusion, we have established nucleophilic‐attack as a broadly effective n‐doping mechanism for OSCs, successfully achieving exceptional conductivity in C_60_ and PC_61_BM using n‐BuLi. Comprehensive mechanistic analyses confirm that n‐BuLi forms covalent bonds with electron‐deficient OSC units, enabling carbanion‐mediated robust n‐doping. This strategy is generalizable across multiple OSCs and extends to other nucleophilic dopants, such as tert‐BuLi and NaOEt. The practical significance is demonstrated by greatly enhanced rectification in organic PN diode devices, attributed to larger built‐in potentials from higher doping strength. Our findings establish a unified chemical‐bonding‐based framework for both n‐ and p‐type doping, overcoming the limitations of conventional redox‐based approaches. Crucially, this mechanistic understanding provides a clear pathway for developing future n‐dopants that are more stable and safer than n‐BuLi. This newfound knowledge paves the way for the rational design and development of next‐generation, high‐performance n‐dopants, ultimately enabling advancements in the performance and efficiency of various organic electronic devices.

## Conflict of Interest

The authors declare no conflict of interest.

## Author Contributions

H.W. performed the characterizations of electrical conductivity as well as the electron spin resonance of different organic semiconductors doped by dopant. Z.G., J.X., C.P., J.D., Y.Z., and W.S. contributed to the device fabrication. C.D. and S.S provide some theoretical calculations for analysis. Y.B. and T.W. provide analysis of mechanism explanation. X.Z, C.C., L.J, L.L., and T.‐Q.N. provided experimental facilities and help reviewed the manuscript. Y.H. conceived the idea and supervised the project. All the authors revised and approved the manuscript.

## Supporting information



Supporting Information

## Data Availability

The data that support the findings of this study are available from the corresponding author upon reasonable request.
