# Supplementary material for: Efficient n‐Doping of Organic Semiconductors via a Broadly Applicable Nucleophilic‐Attack Mechanism
Source: Adv Sci (Weinh). 2025 Nov 6;13(6):e20487. doi: 10.1002/advs.202520487 (PMC12866684; doi:10.1002/advs.202520487)
Supplement: Supplementary file 1 — Supporting Information [file ADVS-13-e20487-s001.docx]

**Supporting Information**

**Efficient n-Doping of Organic Semiconductors via a Broadly Applicable Nucleophilic-Attack Mechanism**

Huan Wei^1,2,3^, Tong Wu^4^, Chuanding Dong^5^, Chen Chen^6^, Zhenqi Gong^1^, Jiangnan Xia^1^, Chengyuan Peng^1^, Jiaqi Ding^1^, Yu Zhang^1^, Wenpei Shi^1^, Stefan Schumacher^5^, Xue Zhang^7^, Yugang Bai^4^, Lang Jiang^8^, Lei Liao^1^, Thuc-Quyen Nguyen^9^ and Yuanyuan Hu^1,2*^

^1^Changsha Semiconductor Technology and Application Innovation Research Institute, College of Semiconductors (College of Integrated Circuits), Hunan University, Changsha 410082, China

^2^International Science and Technology Innovation Cooperation Base for Advanced Display Technologies of Hunan Province, School of Physics and Electronics, Hunan University, Changsha 410082, China

^3^Engineering Research Center for Nanomaterials, Henan University, Kaifeng 475004, China

^4^State Key Laboratory of Chemo/Biosensing and Chemometrics, College of Chemistry and Chemical Engineering, Hunan University, Changsha 410082, China

^5^Department of Physics and Center for Optoelectronics and Photonics Paderborn (CeOPP), Paderborn University, Warburger Strasse 100, Paderborn 33098, Germany

^6^Science and Technology on Advanced Ceramic Fibers and Composites Laboratory, College of Aerospace Science and Engineering, National University of Defense Technology, Changsha 410000, China

^7^Spin-X Institute, School of Microelectronics, South China University of Technology, Guangzhou 511442, China

^8^College of Chemical Engineering, Hebei University of Technology, Tianjin 300401, China

^9^Center for Polymers and Organic Solids, Department of Chemistry and Biochemistry, University of California at Santa Barbara, Santa Barbara, California 93106, United States

Email of the corresponding author: [yhu@hnu.edu.cn](mailto:yhu@hnu.edu.cn)

1. **Experimental Section**

**Materials:** N-DMBI and Chlorobenzene (CB, 99.8%) were purchased from Sigma-Aldrich. N-BuLi (1.6 M in hexanes) was purchased from Damas. Tert-BuLi (1.3 M in pentane) and NaOEt (2.5 M in ethoxide) were purchased from Energy Chemical. [6,6]-Phenyl-C_61_-butyric acid methyl ester (PC_61_BM) was supplied by Xi’an Polymer Light Technology Corp. C_60_, ICBA and acetonitrile were purchased from Aladdin. Hexane, pentane and anhydrous ethanol were purchased from National Pharmaceutical Group Chemical Reagent Co., Ltd. Y6 was purchased from Dongguan Volt-Amp Optoelectronics Tech. Co., Ltd. PDVT-10, PCDTPT, PBBT-4T-2F and N2200 used in this work were synthesized according to the literature.^[1-4]^

**Preparation of Doped Films:** PC_61_BM and C_60_ was dissolved in CB with a concentration of 20 mg/mL and 7 mg/mL, and the solution was stirred at 60 ℃ for one night to allow complete dissolution of the small molecules. For solution doping method, the solutions of host semiconductors and dopant were mixed at desired doping ratios. Mixed solutions were spin-coated on the substrates at 1500 rpm for 20 s. Then, the doped films were thermally annealed. For sequential doping method, the PC_61_BM semiconductor solution was spin-coated at 1500 rpm for 20 s and then the films were annealed at 110 ℃ for 10 min in an inert atmosphere. For C_60_, it was evaporated onto the substrate using a vaporizer at a rate of 0.1 Å/s to a thickness of 30 nm. Next, dopants were applied to the semiconductor at 1500 rpm for 20 s. Among them, n-BuLi, tert-BuLi, NaOEt, and N-DMBI were diluted with hexane, pentane, anhydrous ethanol, and acetonitrile at different dopant concentrations, respectively. It should be noted that nucleophilic reagents need to be evacuated in a small compartment of the glovebox to remove solvents prior to annealing. The whole process took place inside a nitrogen-filled glovebox.

**Preparation of Organic Diodes:** The ITO substrates were ultrasonically cleaned in deionized water, acetone, and isopropyl alcohol each for 15 min, and blown dry by nitrogen gas, and further treated by UV-ozone in the air for 15 min. PEDOT:PSS was spin-coated on ITO under 3000 rpm for 30s and 150 °C for 15 min in air. The P3HT (10 mg/mL) films were deposited on the PEDOT:PSS film by spin-coating at 1500 rpm for 20 s, followed by annealing on hotplate at 130 °C for 5 min in glove box. Then, a 50 nm C_60_ film was deposited by evaporation. Following the preparation method described above for the doped films, C_60_ films doped with N-DMBI and n-BuLi were prepared. Finally, 100 nm-thick Al film was thermally evaporated on top to prepare the top electrode.

**Conductivity Measurement:** For electrical conductivity measurements, the films were deposited on SiO_2_ substrates with predefined electrodes (Cr/Au, 3 nm/35 nm). The conductivity of the prepared devices was measured in glovebox by a four-point probe method through a Keithley 4200 semiconductor analyzer.

**ESR Characterization:** For electron spin resonance (ESR) measurements, the mixed solution was left for 6 hours to fully react, then the sample solutions were dropped into paramagnetic tubes and dried in glovebox to remove the solvent. After sealing the paramagnetic tubes, the ESR spectra was measured on a JEOL JES-FA200 ESR spectrometer at room temperature. For quantitative ESR characterization of doped films, it is guaranteed that the number of molecules in the semiconductor is the same at each doping concentration. Then, the spin density in doped films was estimated by referencing to a standard sample of TEMPO in toluene with known spin density (by assuming that each TEMPO molecule has one spin).

**XPS, NMR, MALDI-TOF MS and Work Function Measurements:** The XPS measurements were performed on film samples by X-ray photoelectron spectrometer (AXIS SUPRA, Japan). ^1^H NMR was recorded on a Bruker AVANCE Neo 400 spectrometer. Matrix-assisted laser desorption/ionization time-of-flight mass spectrometry (MALDI-TOF MS) was performed on a Bruker UltrafleXtreme Mass Spectrometer with α-Cyano-4-hydroxycinnamic acid (CHCA) used as the matrix. For work function measurements, the films were deposited on the Si^++^ substrate and measured on a Kelvin probe (KP020).

**Raman Spectrum Characterization:** The Raman measurements were performed on film samples by Photocurrent Raman Imaging Spectrometer (Witec Alpha300R).

**Statistical Analysis:** The conductivity values are the mean values from at least three samples, with the error bars representing the standard deviation. The data for polaron generation efficiency were obtained from two independent replicate measurements. Mean value was used as the final characterization result for that sample, while the values of the error bars are derived from the population standard deviation, which was specifically calculated using the STDEV.P function in Excel. Data processing was conducted using Origin software.

1. **Supporting Notes**

**Note S1:** Quantitative ESR characterization of doped C_60_ films.

As shown in **Figure S2a**, the double integral of ESR signals of N-DMBI- and n-BuLi-doped C_60_ films significantly increased as the doping level increased. Then, the spin density in doped C_60_ films was estimated by referencing to a standard sample of TEMPO in toluene with known spin density (by assuming that each TEMPO molecule has one spin), with the experimental setting parameter and the Q-factor of the resonator taken into account.^[5]^ The spin numbers of the doped samples were obtained by comparing their second integral of ESR signals with that of the standard sample, according to the equation:

|  | $N=\frac{second integral}{F_{c}*(\sqrt{P_{mw}}*B_{mod}*Q*nB*S*(S+1))}$ | (1) |
| --- | --- | --- |

where B_mod_ is the modulation intensity of magnetic field, nB the Boltzmann population difference, Q the quality factor of the cavity, and S=1/2 for a doublet state.^[6]^ It is apparent that n-BuLi induces more spins (unpaired electrons or polarons) than N-DMBI at the same doping ratio (**Figure S2b**). Polaron generation efficiency (η_i_), defined as the ratio of polarons to dopants in the doped solutions. From the calculation, the maximum η_i_ of 8% was obtained for 60 mol% n-BuLi-doped C_60_ films. In contrast, η_i_ of less than 2% was observed in the N-DMBI-doped C_60_ system, with results shown in **Figure S2c**.

**Note S2:** Characterization of N-DMBI- and n-BuLi-doped N2200

To eliminate the influence of the solvent on the nucleophilic reaction of n-BuLi, we chose sequentially doped N2200 films for ESR testing. First, 9 µL of N2200 solution drop-cast onto a clean glass substrate (3 mm × 10 mm) and allowed to dry into a film. The glass substrate was then immersed in different doping solutions of n-BuLi and N-DMBI for five minutes. Notably, n-BuLi and N-DMBI were diluted with hexane and acetonitrile, respectively. After the immersion doping process, the solvent was removed, and the samples were sealed in ESR tubes for testing, the results are shown in **Figure S15a** and **Figure S15b**. As shown in **Figure S15c**, the double integral of ESR signals of N-DMBI- and n-BuLi-doped N2200 films increased significantly with increasing doping levels. Then, referring to a standard sample of TEMPO in toluene with known spin density, the spin density of the doped N2200 film can be calculated as shown in **Figure S15d**. The polaron density in n-BuLi-doped N2200 films was found to be significantly higher than that in N-DMBI-doped films at comparable doping ratios, suggesting the remarkable doping efficiency of n-BuLi.

1. **Supporting Figures**

**
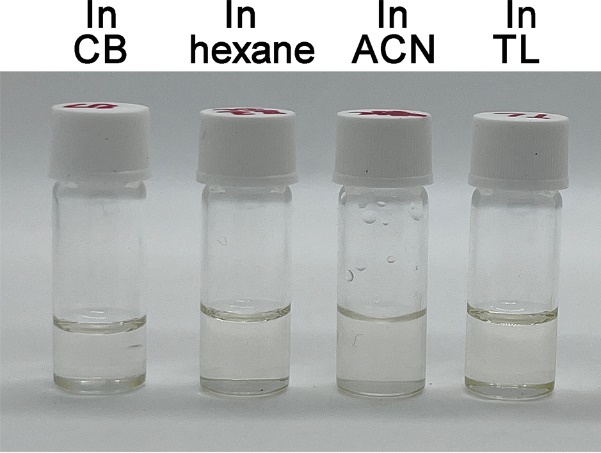
**

**Figure S1.** Solubility of n-BuLi in different solvents (0.2 M), including chlorobenzene (CB), hexane, acetonitrile (ACN) and toluene (TL). It was observed that n-BuLi exhibits excellent solubility in these solvents.

**
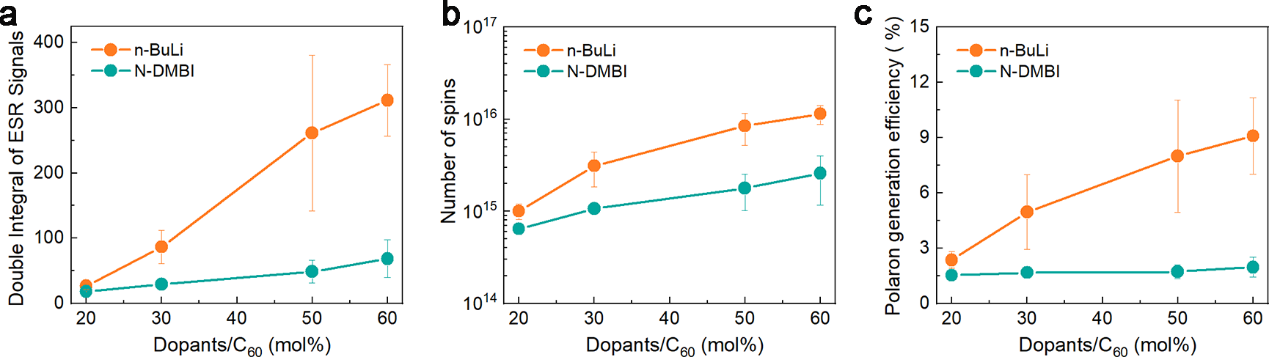
**

**Figure S2.** **(a)** The double integral of ESR signals, **(b)** number of spins and **(c)** polaron generation efficiency of N-DMBI and n-BuLi as a function of doping concentration.


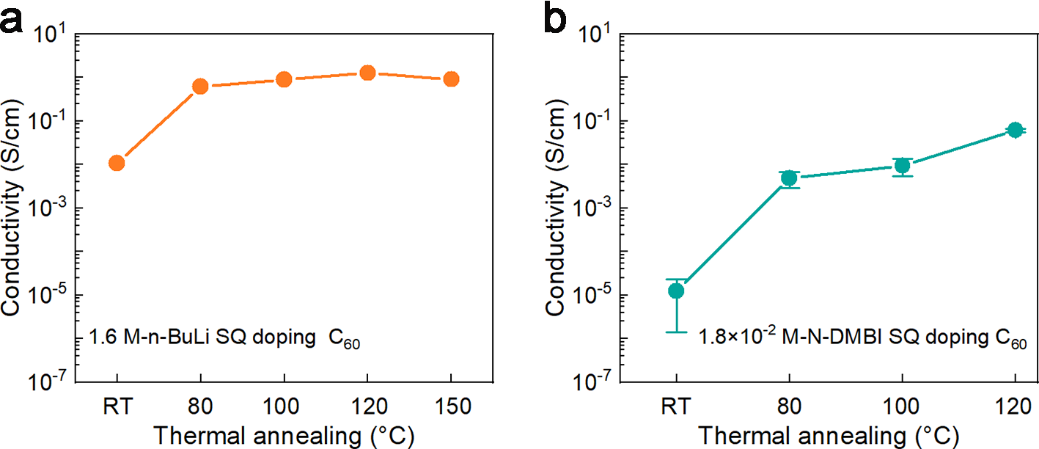


**Figure S3.** Conductivity of the **(a)** n-BuLi- and **(b)** N-DMBI-doped C_60_ films at different annealing temperatures. This indicates that n-BuLi has a superior doping effect than N-DMBI under the same annealing conditions (120 °C).


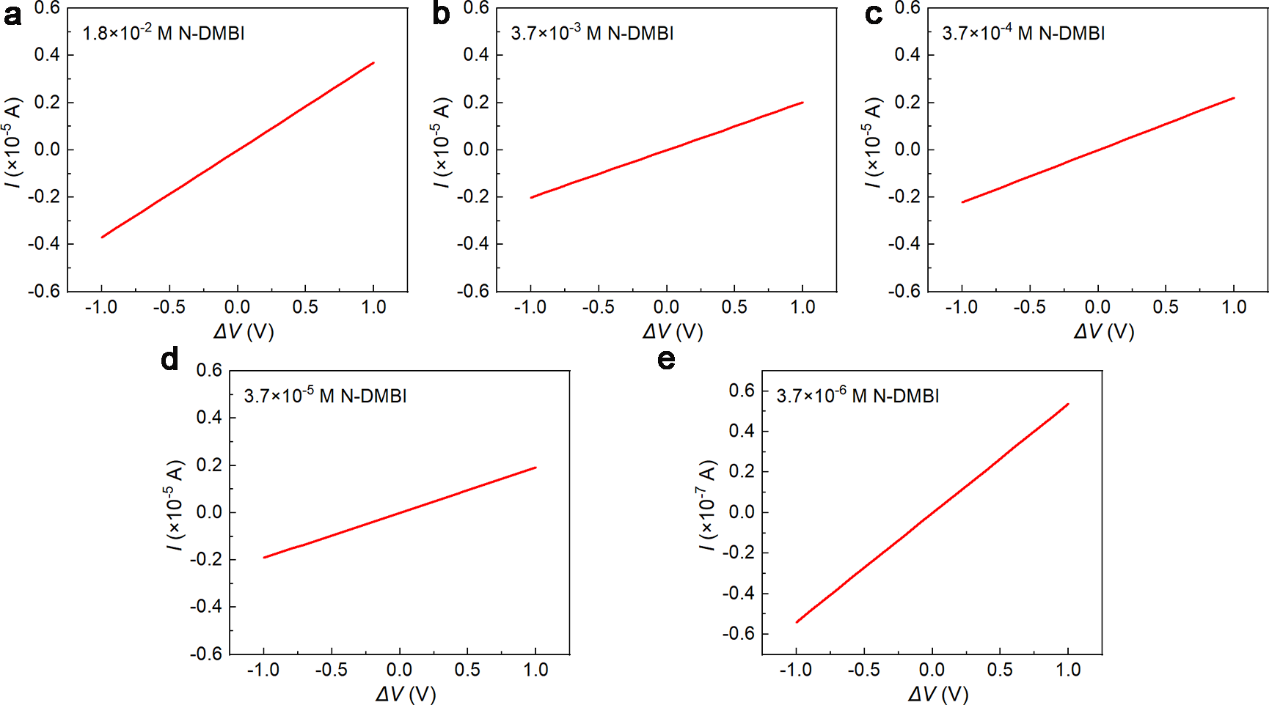


**Figure S4.** Current-voltage curves of C_60_ films with different N-DMBI doping concentrations.


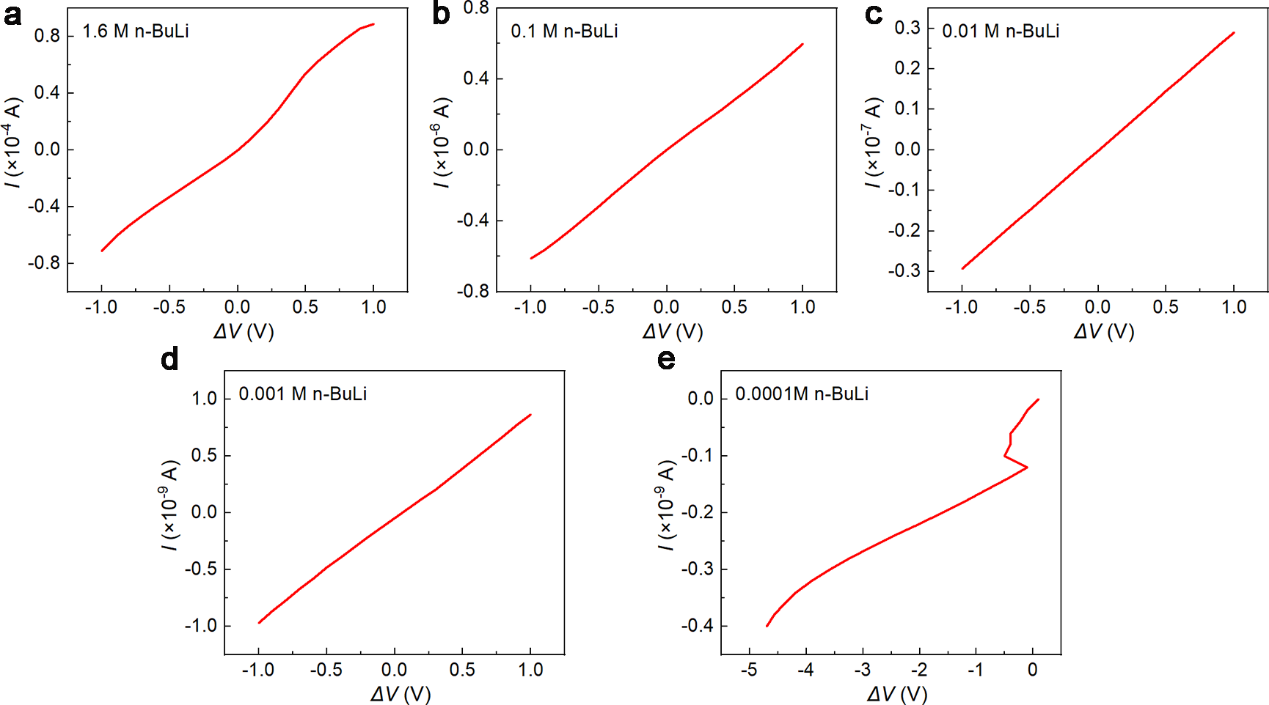


**Figure S5.** Current-voltage curves of C_60_ films with different n-BuLi doping concentrations.


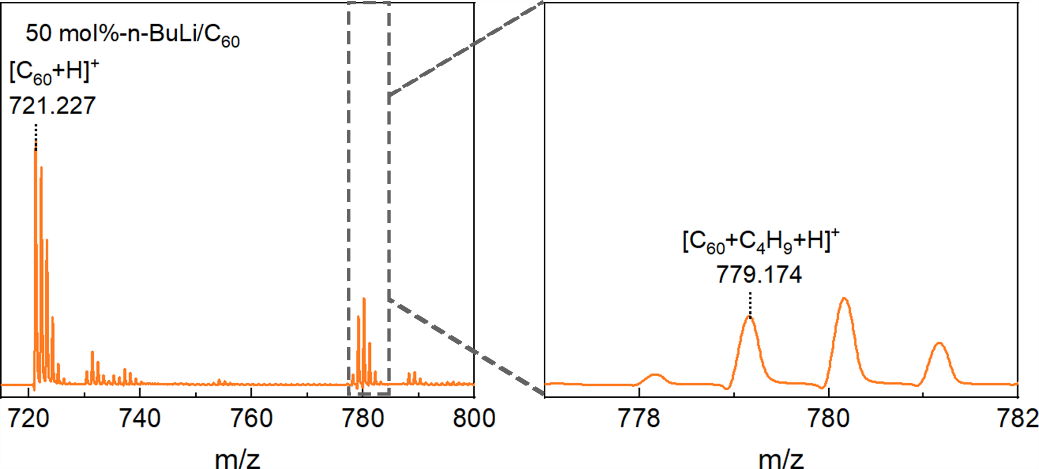


**Figure S6.** MALDI-TOF MS spectrum of the sample containing 50 mol% n-BuLi-doped C_60_. **721.227**: [C_60_+H]^+^ (theoretical most abundant m/z = 721.007, abundance = 100%). **779.174**: [C_60_+C_4_H_9_+H]^+^ (theoretical 2^nd^-most abundant m/z = 779.081, abundance = 69.2%).


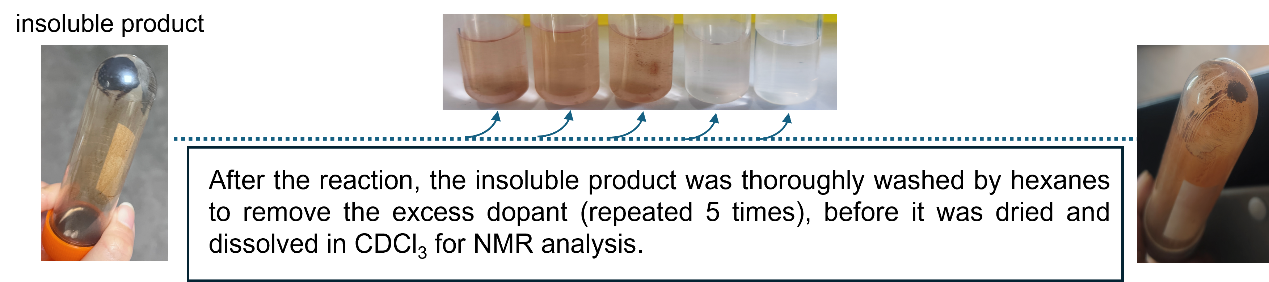


**Figure S7.** Characterization on the ^1^H NMR of the insoluble product by doping C_60_ with n-BuLi.


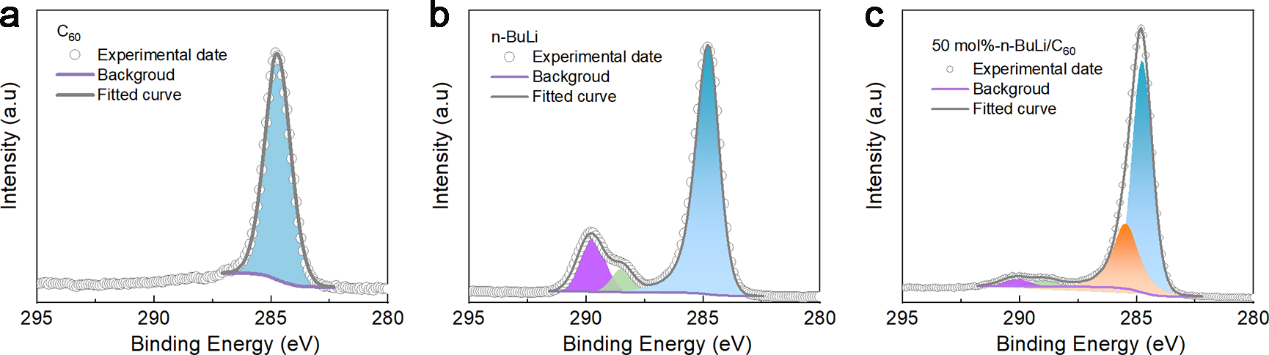


**Figure S8.** The XPS spectra of C 1s in C_60_, n-BuLi and 50 mol% n-BuLi-doped C_60_ films.





**Figure S9.** The Raman spectrum of n-BuLi film, which shows no peaks over the entire test range.


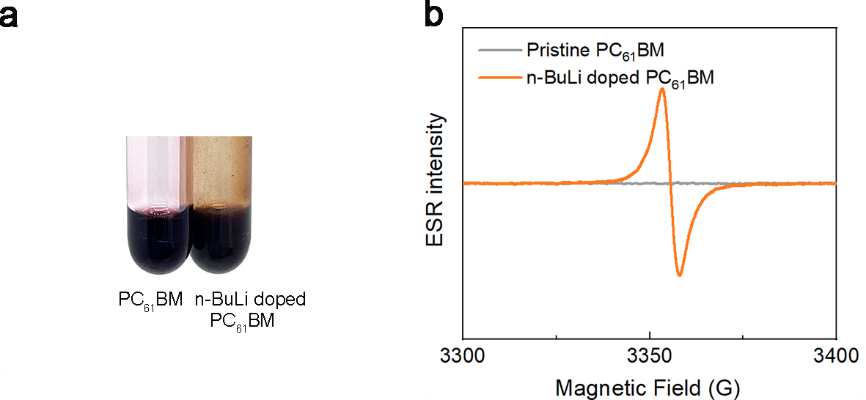


**Figure S10.** **(a)** Color and **(b)** ESR spectra for pristine and n-BuLi-doped PC_61_BM solutions. Color changes and ESR signals were detected in the n-BuLi-doped PC_61_BM solutions, both of which indicate successful doping.


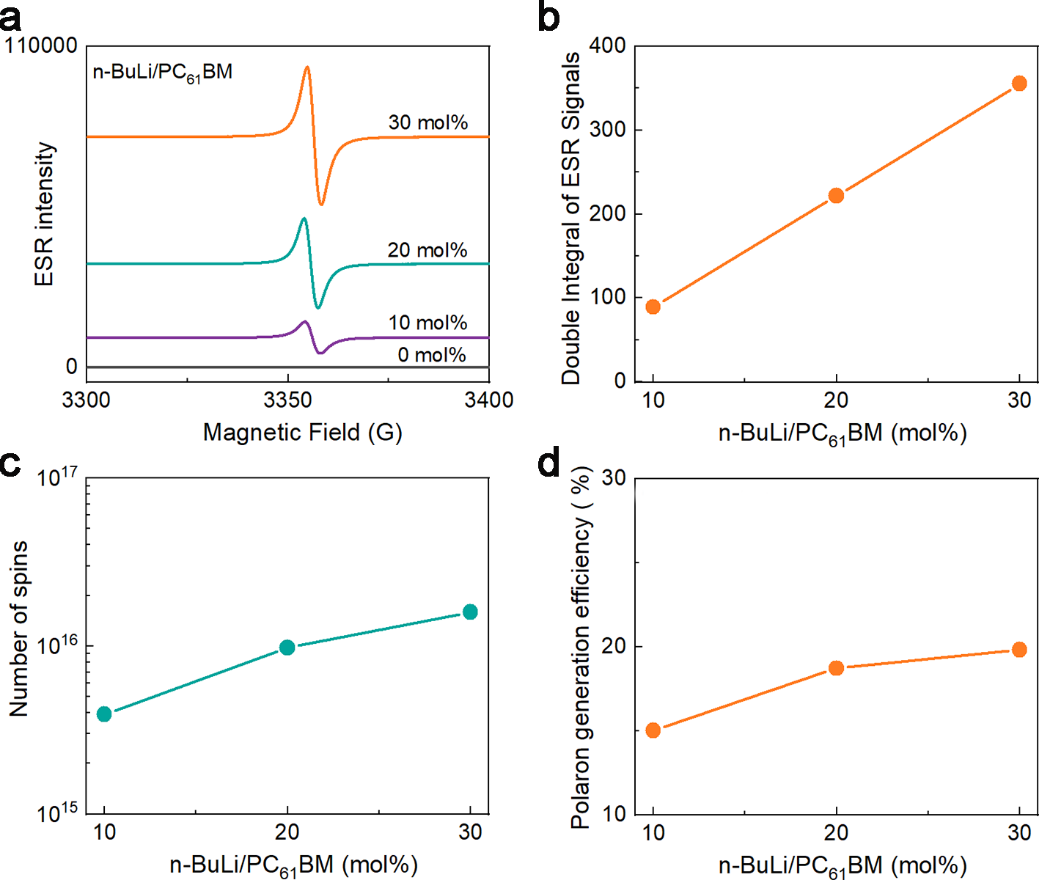


**Figure S11.** **(a)** The ESR spectroscopy of n-BuLi-doped PC_61_BM films as a function of doping concentration measured at room temperature. **(b)** The double integral of ESR signals, **(c)** number of spins and **(d)** polaron generation efficiency of n-BuLi-doped PC_61_BM films as a function of doping concentration. The maximum η_i_ of 20% was obtained for 30 mol% n-BuLi-doped PC_61_BM film. In contrast, η_i_ of less than 16% was observed in the N-DMBI-doped PC_61_BM system,^[4]^ indicating that the stronger doping ability of n-BuLi than N-DMBI.


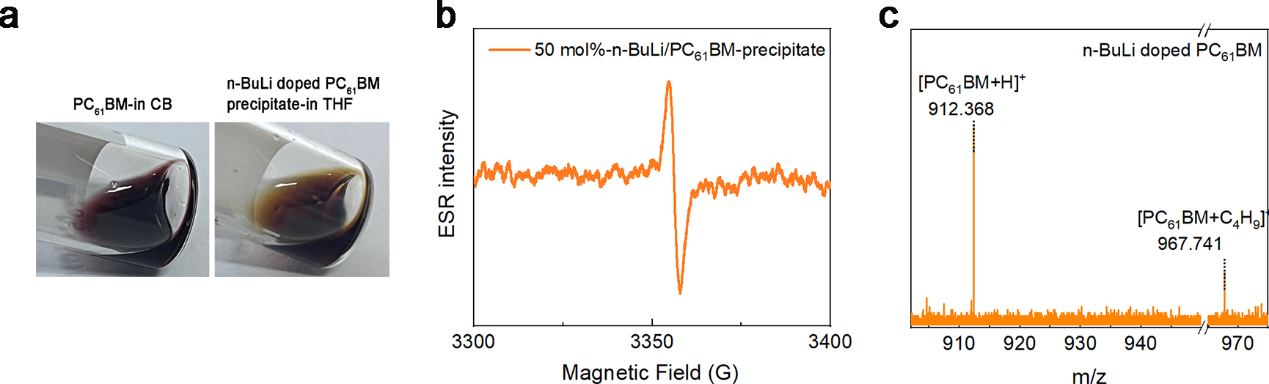


**Figure S12.** **(a)** The re-dissolved precipitate in THF. **(b)** ESR spectra of the n-BuLi-doped PC_61_BM precipitate. **(c)** MALDI-TOF MS spectrum of the n-BuLi-doped PC_61_BM precipitate.


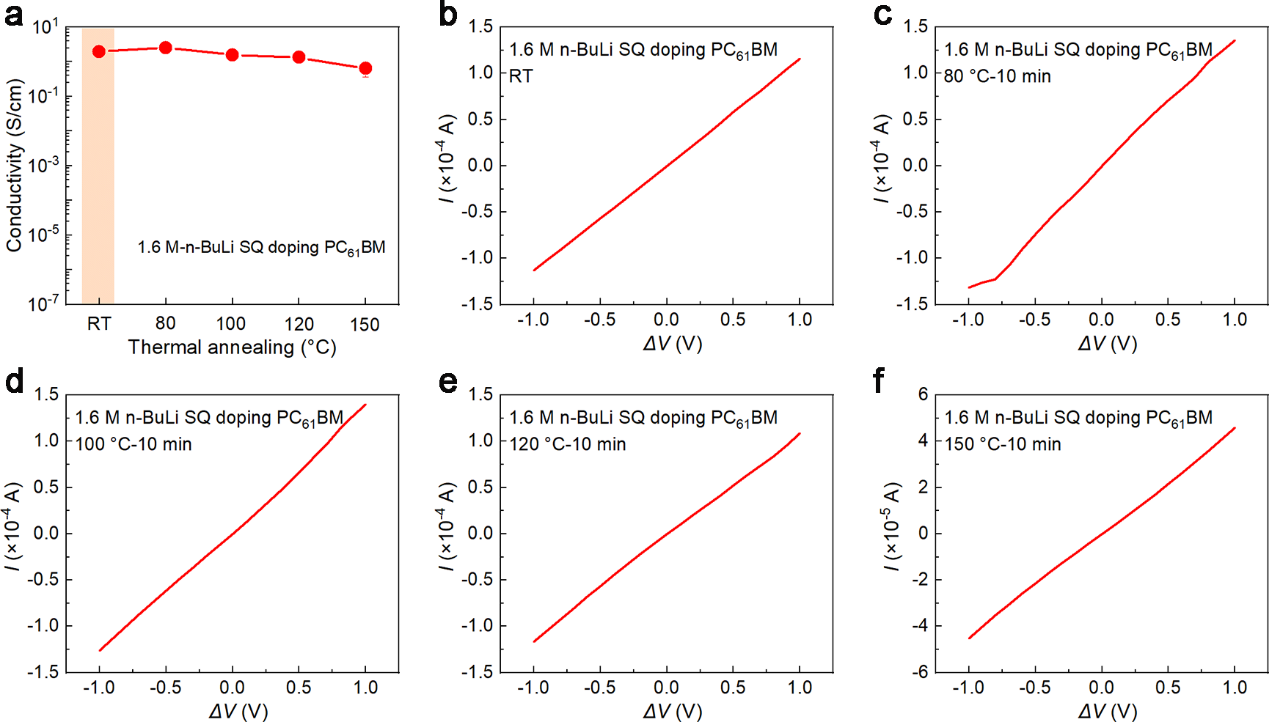


**Figure S13.** Conductivity and current-voltage curves of n-BuLi sequentially doped PC_61_BM films under annealing at different temperatures. The n-BuLi: PC_61_BM system, we choose not to anneal.


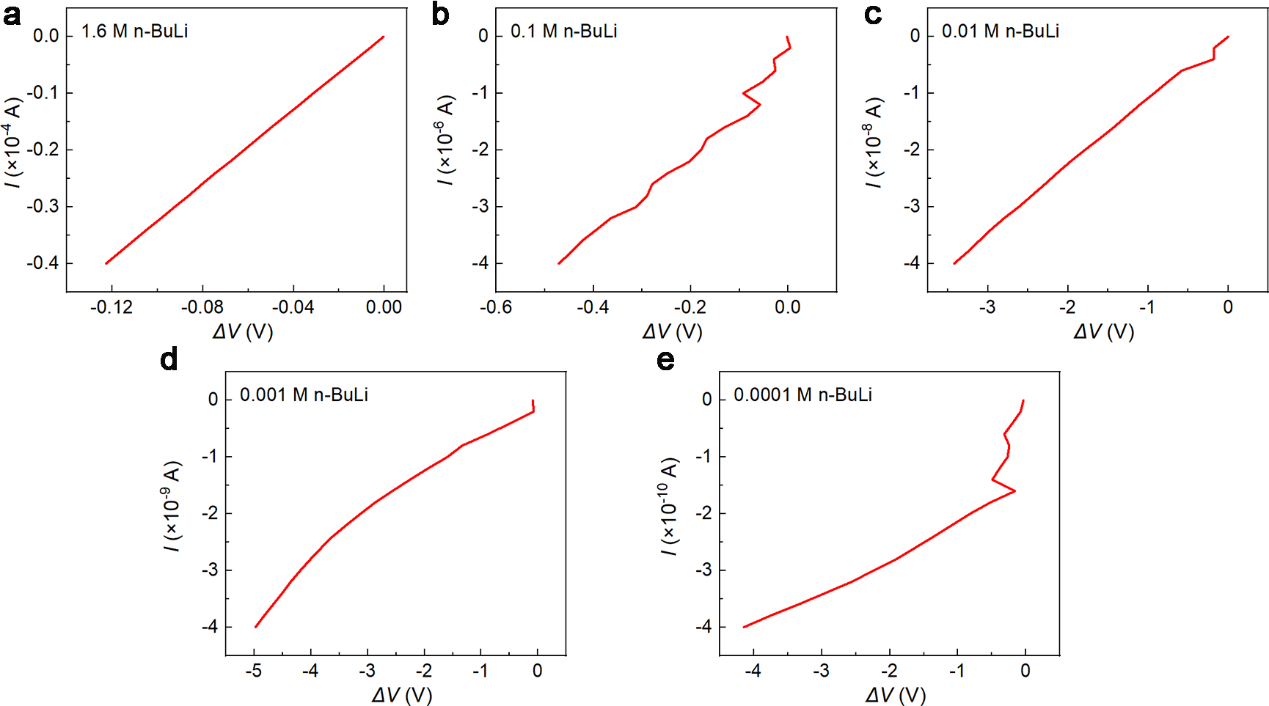


**Figure S14.** Current-voltage curves of PC_61_BM films with different n-BuLi doping concentrations at room temperature.


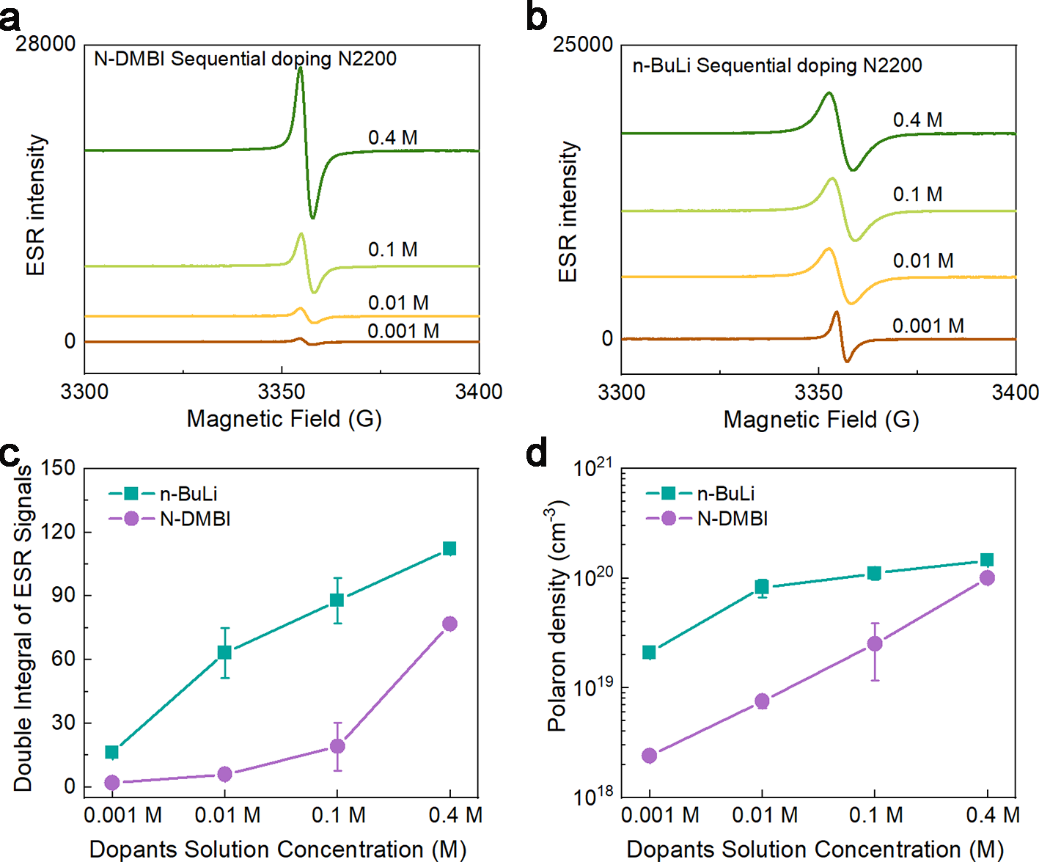


**Figure S15.** The ESR spectroscopy of **(a)** N-DMBI- and **(b)** n-BuLi-doped N2200 films as a function of doping concentration measured at room temperature. **(c)** The double integral of ESR signals and **(c)** polaron density of N-DMBI and n-BuLi as a function of doping concentration.


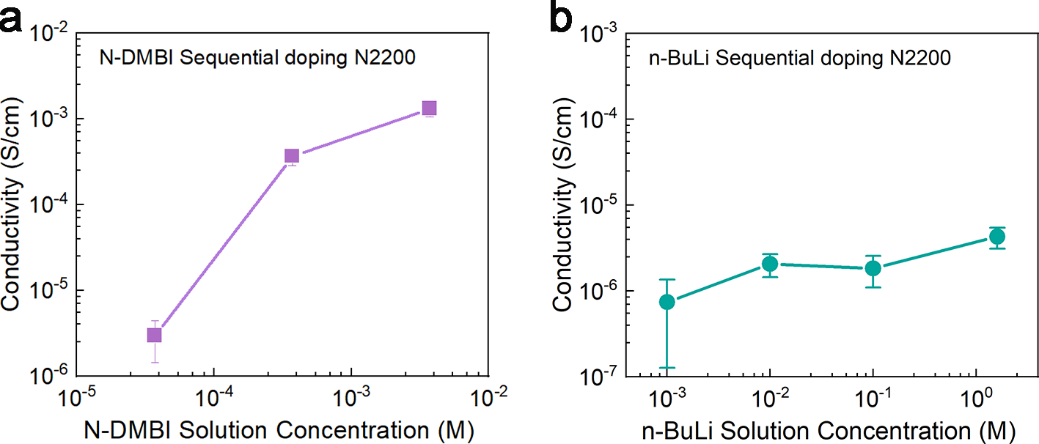


**Figure S16.** The conductivity of **(a)** N-DMBI- and **(b)** n-BuLi-doped N2200 films processed by sequential doping method.

**
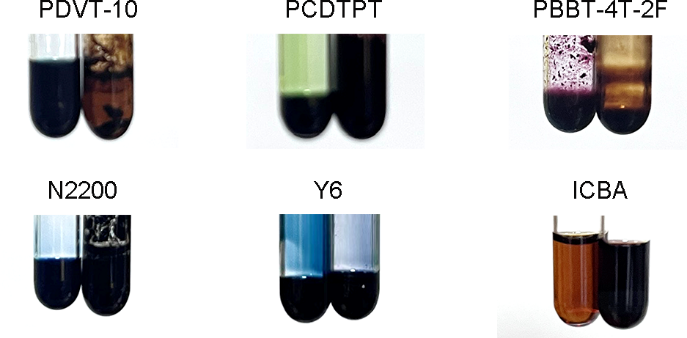
**

**Figure S17.** The comparison of the solutions before and after n-BuLi doping (pristine semiconductor solution on the left and doped solution on the right). The addition of n-BuLi altered the color of these semiconductor solutions, confirming the effective doping of acceptor semiconductors by n-BuLi.

**
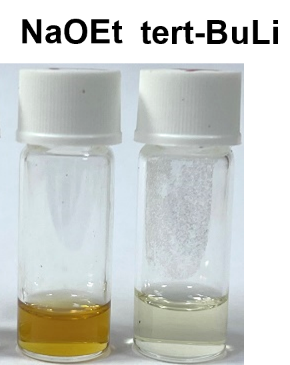
**

**Figure S18.** Photos of NaOEt and tert-BuLi. NaOEt is a dark yellow solution, and tert-BuLi solution shows light yellow color.


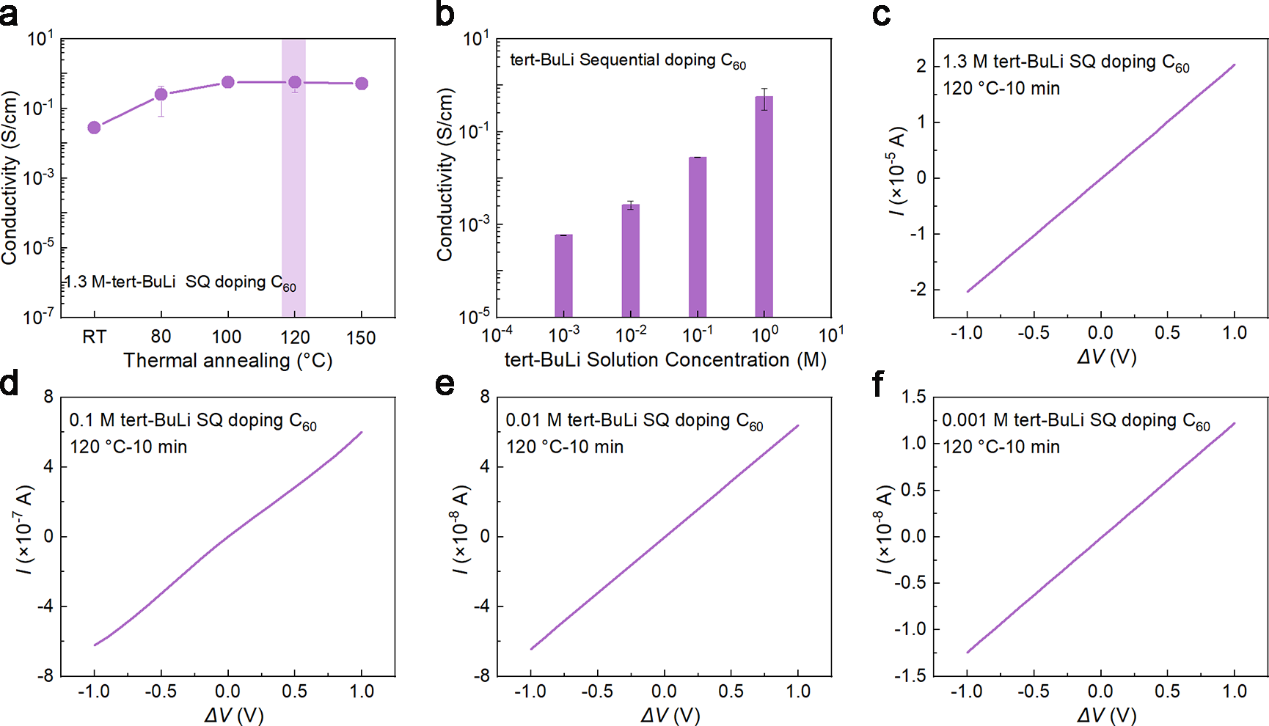


**Figure S19.** **(a)** Conductivity of 1.3 M tert-BuLi sequentially doped C_60_ films under different annealing temperatures. **(b)** Conductivity of C_60_ doped by tert-BuLi as a function of doping concentration. Current-voltage curves of C_60_ films with different tert-BuLi doping concentrations at 120 °C: **(c)** 1.3 M; **(d)** 0.1 M; **(e)** 0.01 M; **(f)** 0.001 M. The channel length and width used here are 160 μm and of 1000 μm, respectively.


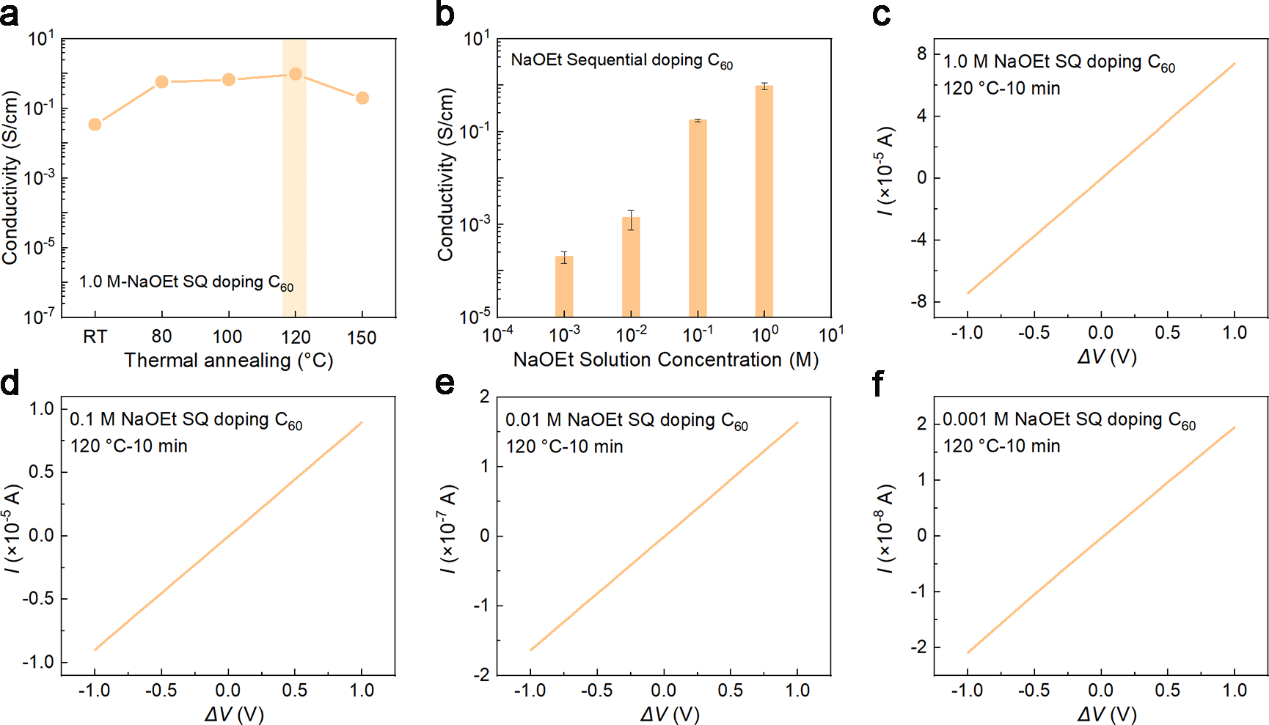


**Figure S20.** **(a)** Conductivity of 1.0 M NaOEt sequentially doped C_60_ films under different annealing temperatures. **(b)** Conductivity of C_60_ doped by NaOEt as a function of doping concentration. Current-voltage curves of C_60_ films with different NaOEt doping concentrations at 120 °C: **(c)** 1.0 M; **(d)** 0.1 M; **(e)** 0.01 M; **(f)** 0.001 M.


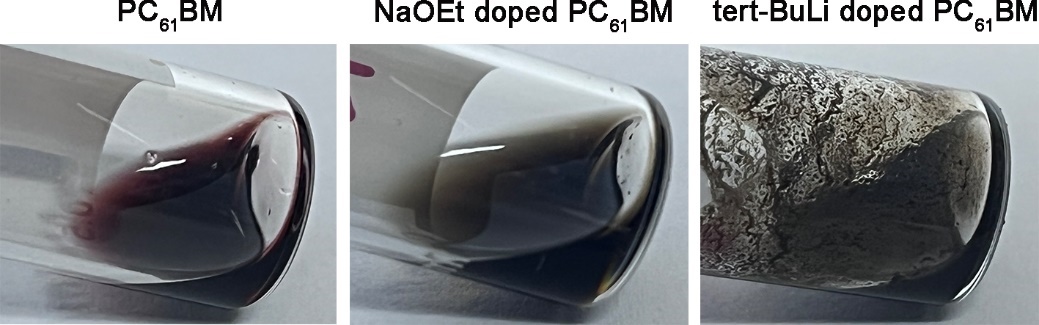


**Figure S21.** Photographs of PC_61_BM solutions before and after doping with NaOEt and tert-BuLi. The color of NaOEt and tert-BuLi-doped solution was observed to become darker.


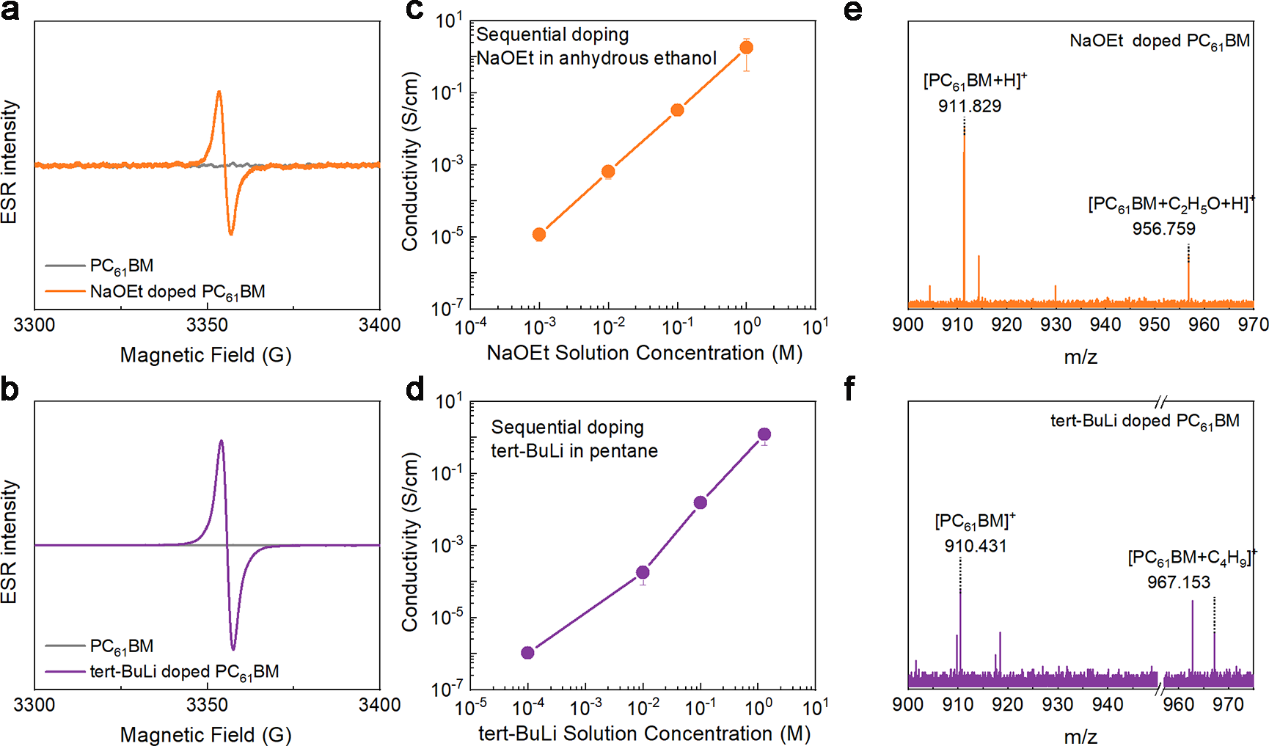


**Figure S22.** The ESR spectroscopy of **(a)** NaOEt- and **(b)** tert-BuLi-doped PC_61_BM. The conductivity of PC_61_BM doped by **(c)** NaOEt- and **(d)** tert-BuLi using sequential doping method. MALDI-TOF MS analysis of **(e)** NaOEt- and **(f)** tert-BuLi-doped PC_61_BM.

**
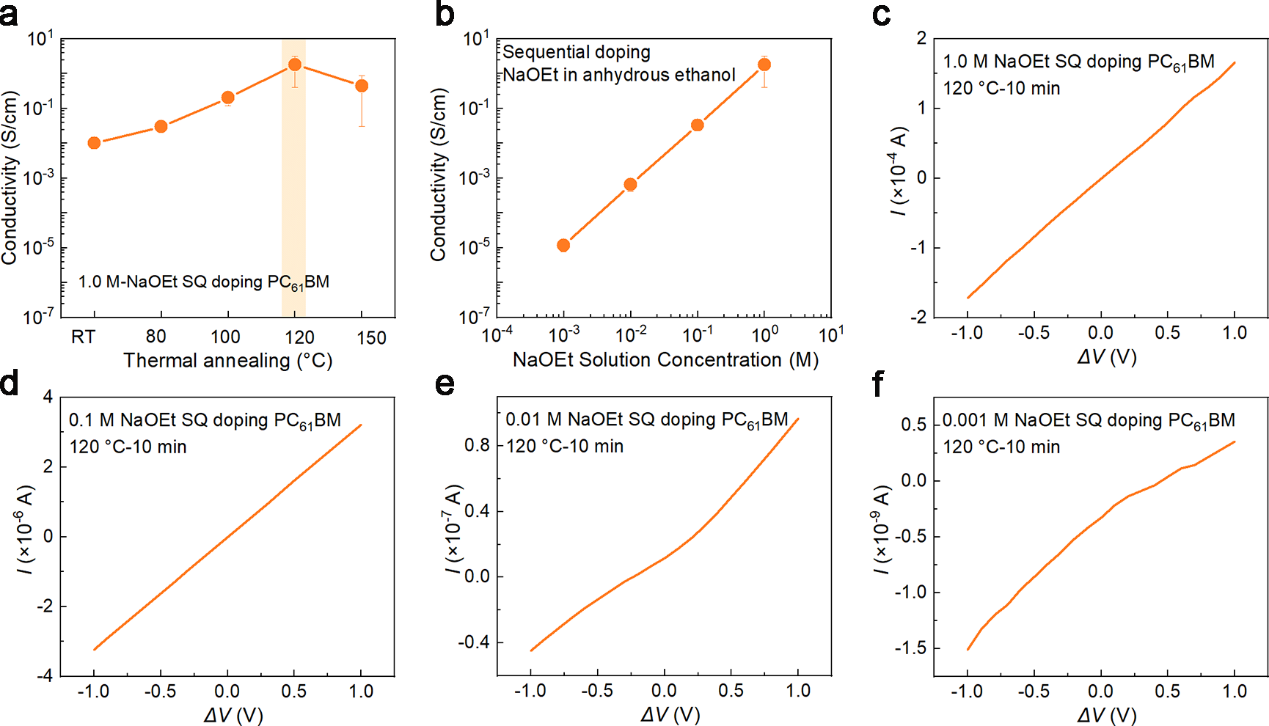
**

**Figure S23.** **(a)** Conductivity of 1.0 M NaOEt sequentially doped PC_61_BM films under different annealing temperatures. **(b)** Conductivity of PC_61_BM doped by NaOEt as a function of doping concentration. Current-voltage curves of PC_61_BM films with different NaOEt doping concentrations at 120 °C: **(c)** 1.0 M; **(d)** 0.1 M; **(e)** 0.01 M; **(f)** 0.001 M. The results suggest that the optimal annealing temperature for the NaOEt: PC_61_BM system is 120 °C.


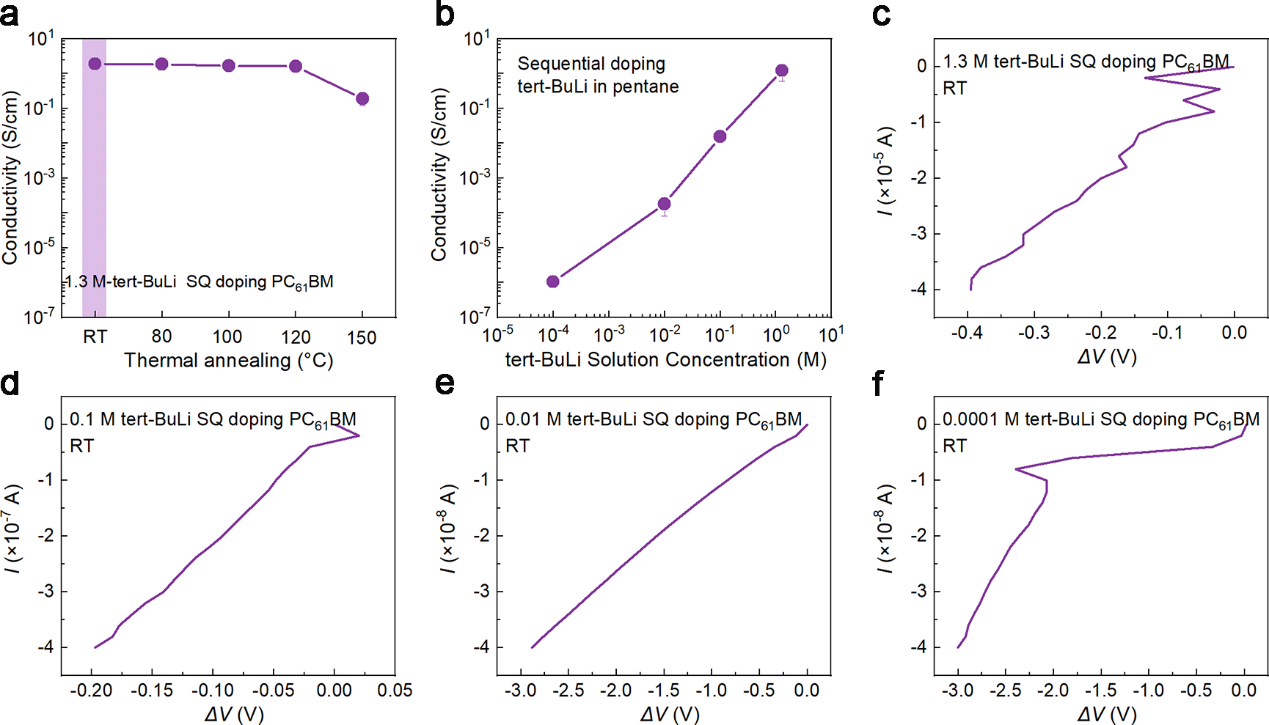


**Figure S24.** **(a)** Conductivity of 1.3 M tert-BuLi sequentially doped PC_61_BM films under different annealing temperatures. **(b)** Conductivity of PC_61_BM doped by tert-BuLi as a function of doping concentration. Current-voltage curves of PC_61_BM films with different tert-BuLi doping concentrations at RT: **(c)** 1.3 M; **(d)** 0.1 M; **(e)** 0.01 M; **(f)** 0.0001 M. The results suggest that the optimal annealing temperature for the tert-BuLi: PC_61_BM system is RT.

**
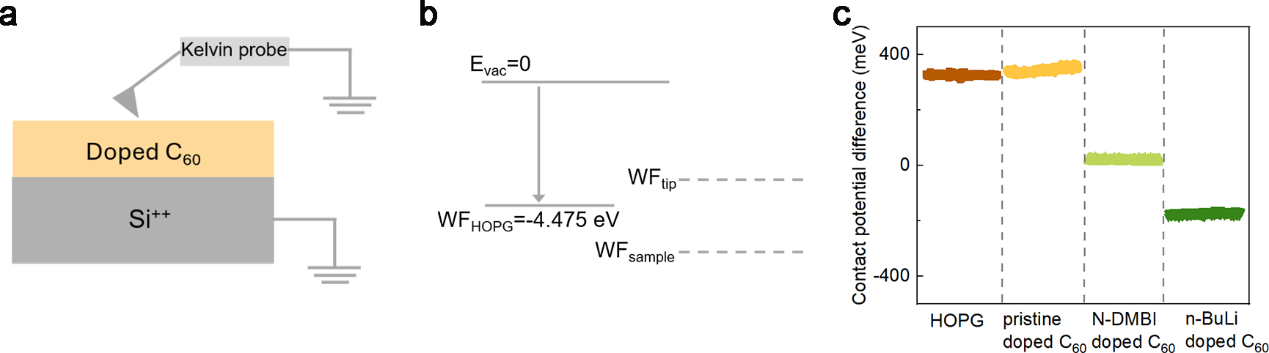
**

**Figure S25.** **(a)** Schematic diagram of Kelvin probe measuring the work function (WF) of doped C_60_ films. **(b)** The energy level diagram shows the detection of WF of the doped C_60_ films by taking the WF highly oriented graphite (HOPG) as a reference. **(c)** The contact potential difference (CPD) between the different samples and the Kelvin probe tip.

**
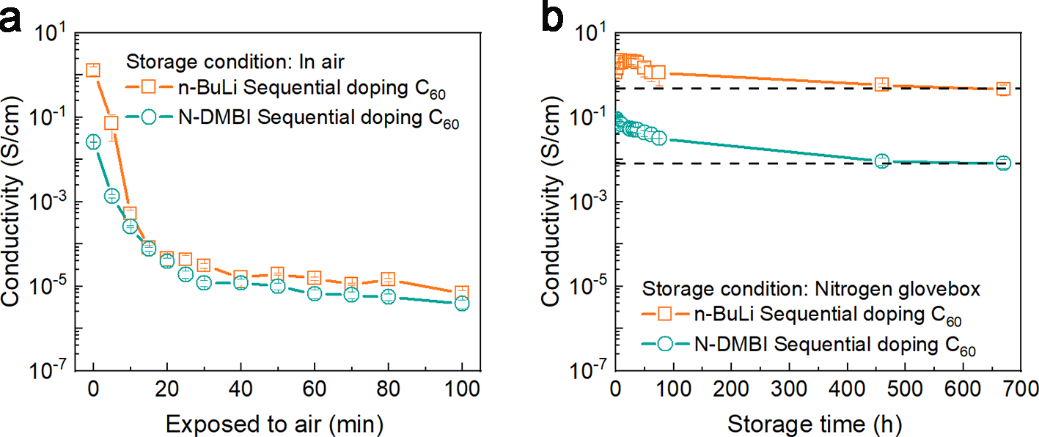
**

**Figure S26.** The stability of conductivity of N-DMBI- and n-BuLi-doped C_60_ films in **(a)** ambient conditions and **(b)** glovebox. The n-BuLi shows very similar stability to N-DMBI in C_60_.

1. **Supporting Tables**

**Table S1:** Summary of literature data for the electrical conductivity of C_60_ doped by different dopants

| OSC | Dopant | Doping  method | Doping  concentration | Conductivity  (S/cm) | Reference |
| --- | --- | --- | --- | --- | --- |
| C_60_ | crystal violet (CV) | / | / | 0.004 | ^[7]^ |
| C_60_ | LCV | Blending | 6.7 mol% | 0.013 | ^[8]^ |
| C_60_ | AOB | Coevaporated | 1.7 mol% | ~0.05 | ^[9]^ |
| C_60_ | (2-Fc-DMBI)_2_ | Coevaporated | 20 mol% | 0.08 | ^[10]^ |
| C_60_ | (2-Rc-DMBI)_2_ | Coevaporated | 18 mol% | 0.1 | ^[10]^ |
| C_60_ | 2-Cyc-DMBI | Blending | 5 mol% | ~3 | ^[11]^ |
| C_60_ | Cr_2_(hpp)_4_ | Blending | 8 mol% | ~4 | ^[12]^ |
| C_60_ | W_2_(hpp)_4_ | Blending | 8 mol% | ~4.5 | ^[12]^ |
| C_60_ | DMBI-POH | Blending | 65 mol% | 5.3 | ^[13]^ |
| C_60_ | o-MeO-DMBI-I | Coevaporated | 14 mol% | 5.5 | ^[14]^ |
| C_60_ | RuCp*(mes)_2_ | Blending | 0.22 | ~8.28 | ^[15]^ |
| C_60_ | (2-Cyc-DMBI)_2_ | Coevaporated | 26 mol% | 12 | ^[10]^ |
| C_60_ | N-DMBI | Sequentially | **0.02 M** | **0.062** | **This work** |
| C_60_ | n-BuLi | Sequentially | **1.6 M** | **1.27** | **This work** |

**Table S2:** Summary of literature data for the electrical conductivity of PC_61_BM doped by different dopants

| OSC | Dopant | Doping  method | Doping  concentration | Conductivity  (S/cm) | Reference |
| --- | --- | --- | --- | --- | --- |
| PC_61_BM | DMBI(H1) | Blending | 0.05 wt% | 1.5 × 10^−6^ | ^[16]^ |
| PC_61_BM | 2,6-Py | Blending | 10 wt% | 2.5 × 10^−5^ | ^[17]^ |
| PC_61_BM | DMBI(H2) | Blending | 0.05 wt% | 5.5 × 10^−5^ | ^[16]^ |
| PC_61_BM | DMBI(H1) | Blending | 0.05 wt% | 6.9 × 10^−4^ | ^[16]^ |
| PC_61_BM | PEI | Vapor | 10 h | 5 × 10^−4^ | ^[18]^ |
| PC_61_BM | DBU | Blending | 1 wt% | 3.6 × 10^−4^ | ^[19]^ |
| PC_61_BM | iPr-TBD | Blending | 10 mol% | ~10^−3^ | ^[20]^ |
| PC_61_BM | TBD | Blending | 10 mol% | ~10^−3^ | ^[20]^ |
| PC_61_BM | CTAB | Blending | 10 mol% | 4.8 × 10^−3^ | ^[21]^ |
| PC_61_BM | TBABr | Blending | 10 mol% | ~5 × 10^−3^ | ^[22]^ |
| PC_61_BM | TBAI | Blending | 10 mol% | 5 .6× 10^−3^ | ^[23]^ |
| PC_61_BM | TBAAcO | Blending | 20 mol% | 3.5× 10^−3^ | ^[23]^ |
| PC_61_BM | TBABr | Blending | 10 mol% | 3.1× 10^−3^ | ^[23]^ |
| PC_61_BM | TBAF | Blending | 10 mol% | 2.4× 10^−3^ | ^[23]^ |
| PC_61_BM | TBAOH | Blending | 10 mol% | 3.9× 10^−3^ | ^[23]^ |
| PC_61_BM | (2-Fc-DMBI)_2_ | Blending | 6 mol% | 1.9 × 10^−3^ | ^[10]^ |
| PC_61_BM | N-DMBI | Blending | 10 wt% | 1.9 × 10^−3^ | ^[24]^ |
| PC_61_BM | AOB | Coevaporation | / | ~10^−2^ | ^[25]^ |
| PC_61_BM | Me-TBD | Blending | 10 mol% | ~10^−2^ | ^[20]^ |
| PC_61_BM | (2-Rc-DMBI)_2_ | Blending | 11 mol% | 1.6 × 10^−2^ | ^[10]^ |
| PC_61_BM | mTPPBr | Blending | 20 mol% | 1.73 × 10^−2^ | ^[22]^ |
| PC_61_BM | N-DMBI | Blending | 30 mol% | 1.16 × 10^−2^ | ^[26]^ |
| PC_61_BM | DPDHP | Blending | 2 wt% | 3× 10^−2^ | ^[27]^ |
| PC_61_BM | (2-Cyc-DMBI)_2_ | Blending | 10 mol% | 4.7 × 10^−2^ | ^[10]^ |
| PC_61_BM | TAM | Blending | / | 4 × 10^−2^ | ^[28]^ |
| PC_61_BM | 2-Cyc-DMBI-H | Blending | 11 mol% | 5.8 × 10^−2^ | ^[10]^ |
| PC_61_BM | DBN | Vapor | 0.5 h | 6.74 × 10^−2^ | ^[29]^ |
| PC_61_BM | 2TBD-C10 | Blending | 10 mol% | 6.5 × 10^−2^ | ^[20]^ |
| PC_61_BM | DMImC | Sequentially | 0.8 mg/ml | 0.1 | ^[30]^ |
| PC_61_BM | FPI | Blending | 20 wt% | 0.13 | ^[31]^ |
| PC_61_BM | NDI-CN/N-DPBI | Inverse-sequential | **/** | 0.4 | ^[32]^ |
| PC_61_BM | P4-t-Bu | Blending | 30 mol% | 1.07 | ^[4]^ |
| PC_61_BM | P2-t-Bu | Blending | 20 mol% | 2.64 | ^[4]^ |
| **PC_61_BM** | **tert-BuLi** | **Sequentially** | **1.3 M** | **1.22** | **This work** |
| **PC_61_BM** | **NaOEt** | **Sequentially** | **1.6 M** | **1.81** | **This work** |
| **PC_61_BM** | **n-BuLi** | **Sequentially** | **1.6 M** | **2.57** | **This work** |

**Supporting References：**

[1] H. Chen, Y. Guo, G. Yu, Y. Zhao, J. Zhang, D. Gao, H. Liu, Y. Liu, *Adv. Mater.* **2012**, 24, 4618.

[2] H. Phan, M. Wang, G. C. Bazan, T. Q. Nguyen, *Adv. Mater.* **2015**, 27, 7004.

[3] Y. Chen, L. Zhao, P.-A. Chen, Y. Li, J. Guo, Y. Liu, X. Qiu, J. Xia, K. Chen, H. Chen, X. Lu, L. Jiang, L. Liao, T.-Q. Nguyen, Y. Hu, *Matter* **2022**, 5, 2882.

[4] H. Wei, Z. Cheng, T. Wu, Y. Liu, J. Guo, P. A. Chen, J. Xia, H. Xie, X. Qiu, T. Liu, B. Zhang, J. Hui, Z. Zeng, Y. Bai, Y. Hu, *Adv. Mater.* **2023**, 35, 2300084.

[5] M. Arvind, C. E. Tait, M. Guerrini, J. Krumland, A. M. Valencia, C. Cocchi, A. E. Mansour, N. Koch, S. Barlow, S. R. Marder, J. Behrends, D. Neher, *J. Phys. Chem. B* **2020**, 124, 7694.

[6] P. Pingel, M. Arvind, L. Kölln, R. Steyrleuthner, F. Kraffert, J. Behrends, S. Janietz, D. Neher, *Adv. Electron. Mater.* **2016**, 2, 1600204.

[7] Z. Berkai, M. Daoudi, N. Mendil, A. Belghachi, *Phys Lett A* **2019**, 383, 2090.

[8] F. Li, A. Werner, M. Pfeiffer, K. Leo, X. Liu, *J. Phys. Chem. B* **2004**, 108, 17076.

[9] N. Hayashi, K. Kanai, Y. Ouchi, K. Seki, *MRS Online Proceedings Library* **2007**, 965, 1303.

[10] B. D. Naab, S. Zhang, K. Vandewal, A. Salleo, S. Barlow, S. R. Marder, Z. Bao, *Adv. Mater.* **2014**, 26, 4268.

[11] C. Gaul, S. Hutsch, M. Schwarze, K. S. Schellhammer, F. Bussolotti, S. Kera, G. Cuniberti, K. Leo, F. Ortmann, *Nat. Mater.* **2018**, 17, 439.

[12] T. Menke, D. Ray, J. Meiss, K. Leo, M. Riede, *Appl. Phys. Lett.* **2012**, 100.

[13] T. Menke, P. Wei, D. Ray, H. Kleemann, B. D. Naab, Z. Bao, K. Leo, M. Riede, *Org. Electron.* **2012**, 13, 3319.

[14] P. Wei, T. Menke, B. D. Naab, K. Leo, M. Riede, Z. Bao, *J. Am. Chem. Soc.* **2012**, 134, 3999.

[15] S. Olthof, S. Mehraeen, S. K. Mohapatra, S. Barlow, V. Coropceanu, J.-L. Brédas, S. R. Marder, A. Kahn, *Phys. Rev. Lett.* **2012**, 109.

[16] Z. Bin, J. Li, L. Wang, L. Duan, *Energy Environ. Sci.* **2016**, 9, 3424.

[17] Y. Jiang, J. Li, S. Xiong, F. Jiang, T. Liu, F. Qin, L. Hu, Y. Zhou, *J. Mater. Chem. A.* **2017**, 5, 17632.

[18] S. Fabiano, S. Braun, X. Liu, E. Weverberghs, P. Gerbaux, M. Fahlman, M. Berggren, X. Crispin, *Adv. Mater.* **2014**, 26, 6000.

[19] L. Hu, T. Liu, J. Duan, X. Ma, C. Ge, Y. Jiang, F. Qin, S. Xiong, F. Jiang, B. Hu, X. Gao, Y. Yi, Y. Zhou, *Adv. Funct. Mater.* **2017**, 27, 1703254.

[20] H. Nakayama, J. A. Schneider, M. Faust, H. Wang, J. Read de Alaniz, M. L. Chabinyc, *Mater. Chem. Front.* **2020**, 4, 3616.

[21] C.-Y. Chang, W.-K. Huang, Y.-C. Chang, K.-T. Lee, C.-T. Chen, *J. Mater. Chem. A.* **2016**, 4, 640.

[22] C. C. Chueh, C. Z. Li, F. Ding, Z. Li, N. Cernetic, X. Li, A. K. Jen, *ACS Appl. Mater. Interfaces* **2017**, 9, 1136.

[23] C.-Z. Li, C.-C. Chueh, F. Ding, H.-L. Yip, P.-W. Liang, X. Li, A. K. Y. Jen, *Adv. Mater.* **2013**, 25, 4425.

[24] P. Wei, J. H. Oh, G. Dong, Z. Bao, *J. Am. Chem. Soc.* **2010**, 132, 8852.

[25] F. Gao, Y. Liu, Y. Xiong, P. Wu, B. Hu, L. Xu, *Front. Optoelectron.* **2017**, 10, 117.

[26] J. Liu, L. Qiu, G. Portale, M. Koopmans, G. Ten Brink, J. C. Hummelen, L. J. A. Koster, *Adv. Mater.* **2017**, 29, 1701641.

[27] K. Shi, Z.-Y. Lu, Z.-D. Yu, H.-Y. Liu, Y. Zou, C.-Y. Yang, Y.-Z. Dai, Y. Lu, J.-Y. Wang, J. Pei, *Adv. Electron. Mater.* **2017**, 3, 1700164.

[28] C. Y. Yang, Y. F. Ding, D. Huang, J. Wang, Z. F. Yao, C. X. Huang, Y. Lu, H. I. Un, F. D. Zhuang, J. H. Dou, C. A. Di, D. Zhu, J. Y. Wang, T. Lei, J. Pei, *Nat. Commun.* **2020**, 11, 3292.

[29] H. Wei, P. A. Chen, J. Guo, Y. Liu, X. Qiu, H. Chen, Z. Zeng, T. Q. Nguyen, Y. Hu, *Adv. Funct. Mater.* **2021**, 31, 2102768.

[30] Y. F. Ding, C. Y. Yang, C. X. Huang, Y. Lu, Z. F. Yao, C. K. Pan, J. Y. Wang, J. Pei, *Angew. Chem., Int. Ed.* **2021**, 60, 5816.

[31] C. Z. Li, C. C. Chueh, H. L. Yip, F. Ding, X. Li, A. K. Jen, *Adv. Mater.* **2013**, 25, 2457.

[32] G. Zuo, Z. Li, E. Wang, M. Kemerink, *Adv. Electron. Mater.* **2018**, 4, 1700501.
